# Patterns of species richness and the center of diversity in modern Indo-Pacific larger foraminifera

**DOI:** 10.1038/s41598-018-26598-9

**Published:** 2018-05-29

**Authors:** Meena Förderer, Dennis Rödder, Martin R. Langer

**Affiliations:** 1 Steinmann Institute for Geology, Mineralogy and Paleontology, Department of Earth Sciences, University of Bonn, Nussallee 8, 53115 Bonn, Germany; 2 Herpetology Section, Zoological Research Museum Alexander Koenig, Adenauerallee 160, 53113 Bonn, Germany

## Abstract

Symbiont-bearing Larger Benthic Foraminifera (LBF) are ubiquitous components of shallow tropical and subtropical environments and contribute substantially to carbonaceous reef and shelf sediments. Climate change is dramatically affecting carbonate producing organisms and threatens the diversity and structural integrity of coral reef ecosystems. Recent invertebrate and vertebrate surveys have identified the Coral Triangle as the planet’s richest center of marine life delineating the region as a top priority for conservation. We compiled and analyzed extensive occurrence records for 68 validly recognized species of LBF from the Indian and Pacific Ocean, established individual range maps and applied Minimum Convex Polygon (MCP) and Species Distribution Model (SDM) methodologies to create the first ocean-wide species richness maps. SDM output was further used for visualizing latitudinal and longitudinal diversity gradients. Our findings provide strong support for assigning the tropical Central Indo-Pacific as the world’s species-richest marine region with the Central Philippines emerging as the bullseye of LBF diversity. Sea surface temperature and nutrient content were identified as the most influential environmental constraints exerting control over the distribution of LBF. Our findings contribute to the completion of worldwide research on tropical marine biodiversity patterns and the identification of targeting centers for conservation efforts.

## Introduction

Warm-water coral reefs are exceptionally diverse ecosystems that are home to more than three million species^1^. Reefs of Southeast Asia have been identified as the most extensive (73,000 km^2^) and diverse of the world^2^ but are also among the most vulnerable^3^, since coral cover is rapidly declining^4^. About 75 percent of the world’s reefs are currently threatened^3^ by rising temperatures, climate change, and direct human perturbations^1^. This applies particularly to the Philippines that are facing high population pressures coupled with severe exploitation of marine resources^5^.

**Figure 1:**
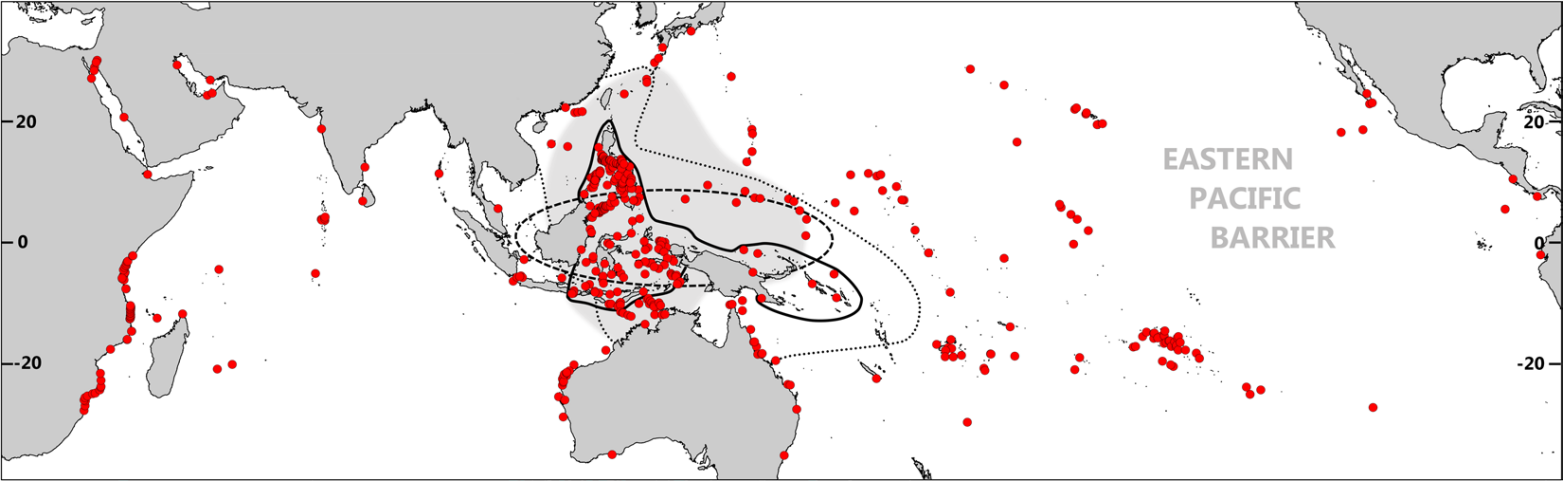
Distribution of occurrence records. Map showing sample locations of all Larger Benthic Foraminifera (LBF) species point data included in this study (red dots). The solid line marks the delineation of the Coral Triangle (after Veron *et al*.^2^). The dashed line delineates the generic center of diversity for LBF as identified by Belasky^19^. The extent of the inner, Central Indo-Pacific province for LBF as identified by Langer & Hottinger^13^ is highlighted in light grey, the dotted line reflects the center of generic LBF alpha diversity as delineated by Renema *et al*.^26^. The map was generated in ArcMap 10.3.1 (https://www.esri.de/support-de/produkte/arcgis-for-desktop-10-3) and modified in Adobe Photoshop CS6 (http://www.adobe.com/de/products/catalog.html).

Mapping large-scale biogeographic patterns is vital for setting conservation priorities by revealing biogeographical variability and enabling the identification of species richness hot- and coldspots. It can further provide insights into underlying mechanisms that promote richness patterns^6^. The best explored and resolved biogeographic patterns in the Indo-Pacific are those of corals and shore fish^7–9^. To date, a total of 627 species of scleractinian corals, representing 74 percent of all coral species worldwide, have been identified in the Coral Triangle^7^. The Coral Triangle is defined as a roughly triangular area that includes seascapes of the Philippines, Malaysia, Indonesia, Papua New Guinea, the Solomon Islands and Timor-Leste (Fig. 1). It has been delineated primarily by coral species distribution and richness with at least 500 species being present within each of the 16 identified ecoregions^2^. Exceptional coral diversity has recently been further identified in the Sunda Shelf Ecoregion and at lower mesophotic depths of the Coral Sea and the Great Barrier Reef^7,10^.

Combined species-level richness maps revealed that habitat-forming taxa (corals, mangroves, seagrasses) are most diverse in the western part of the Coral Triangle (Philippines and large parts of eastern and southern Indonesia)^11,12^, while the central Philippines appear to represent the bullseye of the overall richness pattern^12^. Research on the exact delineation, dimension, and position of the diversity center is still ongoing and necessitates additional distribution data of a wider array of model taxa^6^.

Benthic foraminifera are ideal model taxa for biogeographic studies, as they represent the most diverse group of marine testate protists, have an excellent geological and modern biogeographic record and are globally distributed from marginal to deep-sea environments^13^. Shallow-water benthic foraminifera from the tropical, subtropical, and temperate Indo-Pacific have been extensively studied since the 1800s and were later complemented by comprehensive surveys from previously largely unexplored areas (see List S1).

Benthic foraminifera show a broad variety of feeding mechanisms including a mixotrophic lifestyle based on the symbiosis with photosynthetic microalgae. Symbiont-bearing taxa, commonly referred to as Larger Benthic Foraminifera (LBF), constitute a polyphyletic functional group that is highly adapted to oligotrophic conditions and restricted to the photic zone in warm waters between latitudes 40°N and 31°S^13,14^. They are most abundant in carbonate-rich environments of shelf areas in modern tropical oceans where they contribute substantially to reefal accretion and substrate stability^14–18^.

Previous biogeographic analyses on benthic foraminifera have been conducted on morphospecies as well as on genetic phylotypes^13,19–21^. Large-scale studies on latitudinal diversity gradients have been performed on Atlantic deep-sea foraminifera^22^ but not on LBF. Biogeographic studies on generic-level show that LBF diversity is highly correlated to that of scleractinian corals^19^ and assumed to peak within the Indo-West Pacific where it was established during the Miocene^13,23–26^. The center of diversity in Indo-Pacific LBF, however, remains ambiguous.

Species distribution modeling (SDM) has become a useful tool to predict and quantify the distribution of taxa in geographic space with applications in such diverse fields as setting up conservation priorities, testing biogeographic hypotheses or assessing the impact of human-induced perturbations^27,28^. Here, we apply an overlay of single SDMs using MaxEnt and, for comparison, an overlay of minimum convex polygon range maps to identify the center of species richness in Indo-Pacific LBF. The species distribution data is based on occurrence records of 68 LBF species which we identified and synonymized for the Indian and Pacific Ocean. This first evaluation on species-level aims to contribute to the completion of the overall picture of global tropical marine biodiversity patterns and helps to refine the delineation of high-priority areas for conservation.

## Material and Methods

### Species records

Sixty-eight validly recognized species of LBF were identified and synonymized for the Indian (including the Red Sea and the Persian Gulf) and Pacific Ocean. These include 3 species within the Alveolinidae, 21 species within the Peneroplidae, 9 species within the Soritidae, 6 species within the Amphisteginidae, 14 species within the Calcarinidae, and 15 species within the Nummulitidae (Table S1). To date, 32 of them have been recognized by molecular analyses^29^ but not all of them have been examined from their type localities. Several LBF species are particularly rare or endemic and have not yet been subjected to molecular analysis. There is also still disagreement about the number of valid subfamilies, genera, and species among researchers^30,31^. In general, benthic foraminiferal diversity is presumed to be rather under- than overestimated^20,32^. Several studies have reported high species richness (>30 taxa of LBF) from localities in the Central Indo-Pacific (Table S2) and a total of 40 species (of which 37 were included in this study) were recorded from around New Caledonia alone^33^.

A number of 2,964 occurrence records from 507 sample sites were included for establishing the richness maps (Table S2). The investigated sample sites cover a latitudinal range between 33°N and 34°S (Fig. 1). The point data compiled for this study include extensive primary sources, revisions on species level for generic studies^34^ and records from the scientific literature (List S1). Primary data are from original field studies conducted by the authors and from own unpublished data sets. The total data set includes comprehensive species-level records covering the full range of tropical and subtropical reefal, lagoonal and shallow shelf habitats within a general depth range down to the limits of the photic zone (<150 m).

The bulk of distributional data have been carefully extracted from a total of 114 literature studies published by generations of micropaleontologists between 1826 and 2017. Because species-level taxonomy may vary from author and significantly affects biogeography, all literature sources have been critically reevaluated by the authors. The literature records were then synonymized, provided that species were adequately illustrated and key features were clearly recognizable or were as such in previous studies of the respective author (Table S3). Ambiguous species and generic records were not taken into account.

### Mapping procedure

Individual grid-based range maps of the Indo-Pacific symbiont-bearing larger foraminifera species were established and subsequently combined to richness maps. All data for creating the maps and richness gradients were processed in R (https://cran.r-project.org/). Two different methods have been applied in order to improve the informative value: (1) an overlay of individual Minimum Convex Polygon (MCP) range maps with no further consideration given to habitat suitability and (2) an overlay of species ranges as predicted by Species Distribution Models (SDMs). Both richness maps are hybrids as species occurrences that allowed no SDM performance or creation of MCPs were included subsequently by buffering and merging each point data with a 500 km radius. The maps were created using ArcMap 10.3.1 for Desktop and projected onto the WGS 1984 PDC Mercator coordinate system centered at 180° longitude. The maps have a 2.5-arcminute (of a longitude/latitude degree) spatial resolution, equivalent to about 4.5 km at the equator. In order to quantify potentially colonizable areas, Coral Reef regions layers from *The IUCN Red List of Threatened Species*™ were buffered with a 100 km radius and used as a mask, restricting the projection and prediction of the species ranges on areas LBF are generally distributed. The georeferenced landscape is a 1:10 m scale and was obtained from *Natural Earth* free vector and raster map data.

For generating the MCP overlay map, polygons were created for 55 of the 68 species identified. The remaining 13 species were represented by less than three occurrence records and were included subsequently as mentioned above (Table S2).

### Species Distribution Modeling (SDM) computation

We used MaxEnt software version 3.3.3k. MaxEnt is a “Maximum Entropy” algorithm software that is one of the most popular SDM computing programs and has been already successfully applied on benthic foraminifera^35–37^. MaxEnt is a powerful grid-based machine learning method that works with presence/background data, contrasting the given set of presence data with a random set of background points from all over the study area^38^. For SDM training, we used the environmental data from the coral reef region areas. The functional principle of an entropy maximization algorithm is that it initially assumes a uniform probability within the geographic space and successively adapts and restricts the distribution till it fits the given input data of occurrence records and environmental variables^39^. For applications in geographic information systems, the program requires the point data from where a particular species was recorded, a georeferenced land- or seascape, and an environmental variable data set.

Out of the 68 species identified, the occurrence data of 52 allowed a modeling performance (Table S2). The remaining 17 species were included by buffering and merging each point data with a 500 km radius. The potential distributions of the species were modeled using environmental variables from Bio-ORACLE (ocean rasters for analysis of climate and environment), which has been specifically designed for modeling marine species distributions^40^.

The original set of environmental variables comprised calcite concentration (mol/m^2^; calcite), pH (ph), dissolved oxygen (ml/l; dissox), phosphate (µmol/l; phos), nitrate (µmol/l; nitrate), salinity (PSS), silicate (µmol/l; silicate), as well as chlorophyll a concentration (mg/m^3^; annual monthly min, max, mean and range), cloud fraction (%, annual monthly max, mean and min), diffuse attenuation coefficient at 490 nm (m^−1^; annual monthly min, max and mean); photosynthetically available radiation (Einstein/m^2^/day; annual monthly maximum and mean), and sea surface temperature (°C; annual monthly min, max, mean and range).

Based on 10,000 randomly chosen unique grid cells all environmental variables were extracted and a principal component analysis was performed in order to remove potential multi-collinearity and to reduce the number of predictors. Principal components were subsequently projected into geographic space resulting in six PCs with Eigenvalues >1, which were used for SDM development (Table S4).

For SDM computation and evaluation, ten different models were computed for each species, each trained with 80% of the species records used for model training and 20% used for model evaluation using a bootstrap approach. All replicates were subsequently merged for further processing and the resulting probability surfaces were turned into binary presence/absence maps using the *equal sensitivity and specificity threshold* as the minimum threshold above which the species is considered to be present.

The interpretation and predictive performance of the model can be evaluated through the logistic output that MaxEnt provides per default. The Area Under the Receiver Operating Characteristic (ROC) Curve (AUC) is a common measure of model accuracy^41^. Its value can be interpreted as the probability that an occurrence record would be identified as such. AUC values range between 0 (model performance worse than random) over 0.5 (no better than random) to 1 (perfect discrimination). AUC values greater than 0.9 show very good, greater than 0.8 show good and greater than 0.7 show useful discrimination ability of the model^41^. For the evaluation of the model performance, it is referred to the AUC test values that are given for each of the species involved. For more details on the operating mode of MaxEnt and the interpretation of its output see Elith *et al*.^42^.

As SDM predicts the occurrence of species solely based on selected abiotic environmental variables, occurrences have been provided with a buffer of 2,500 km for selected species with limited distribution ranges (Table S2).

The SDM output was subsequently used for creating boxplots in a 3° resolution over latitudinal and longitudinal grid space. These boxplots were then merged into area charts to visualize richness/suitability gradients for LBF in the Indo-Pacific realm.

### Data availability

The datasets generated during and/or analyzed during the current study are available from the corresponding author on reasonable request.

## Results

**Figure 2:**
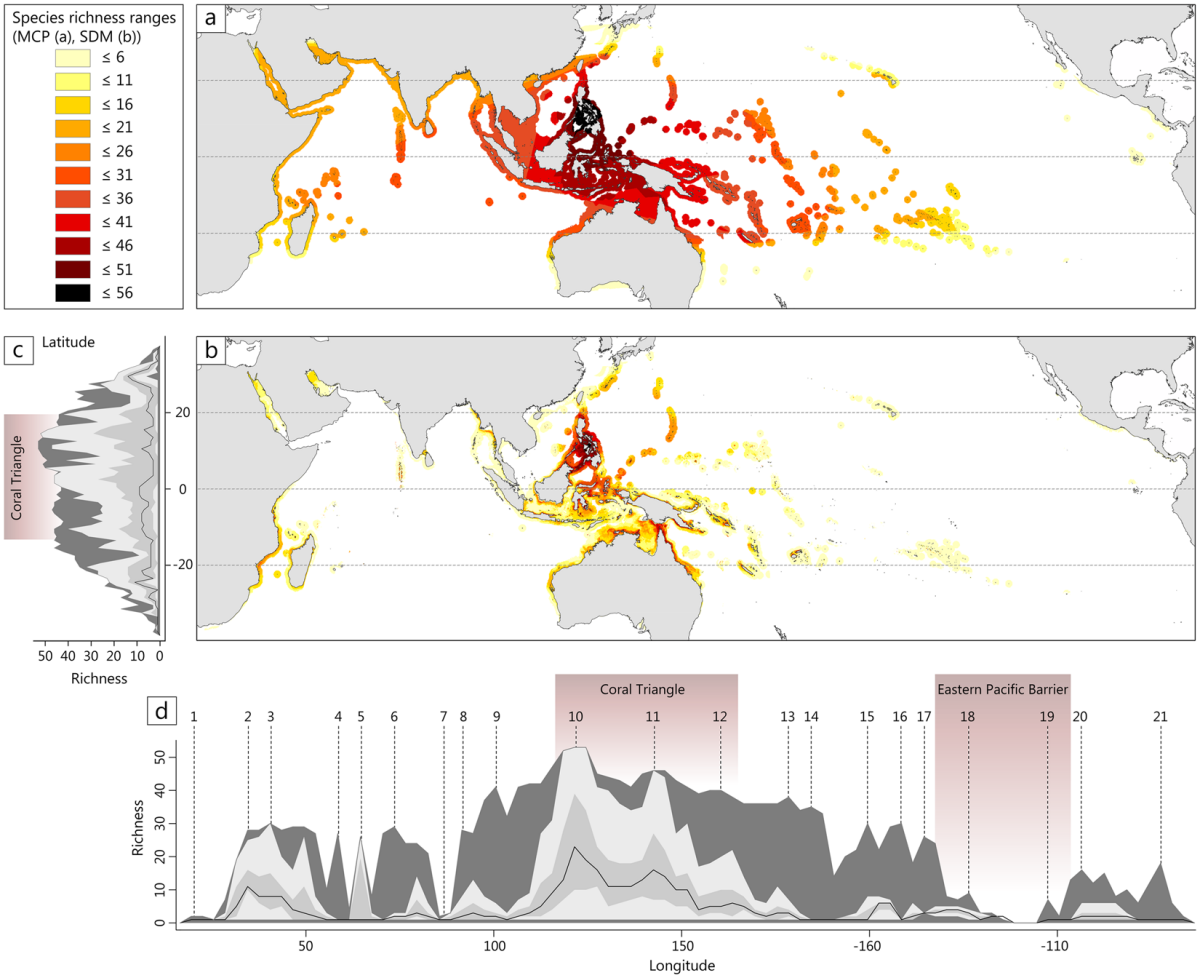
Richness patterns for Indo-Pacific symbiont-bearing larger foraminifera. (**a**) MCP richness map and (**b**). SDM richness map. Legend with corresponding colors of species richness ranges for both maps in the upper left corner. (**c**) Latitudinal and (**d**) longitudinal distribution of SDM richness based on single boxplots in 3° resolution. Corresponding colors: dark grey = maximum richness values, medium grey = quartiles, light grey = whiskers, solid line = median. The numbers in d referring to i.a.: (1) South Africa, (2) Red Sea and Mozambique Channel, (3) Tanzania and northern Mozambique Channel, (4) Mascarenes and Seychelles, (5) Rodrigues Island, (6) Maldives, (7) Bay of Bengal, (8) Andaman Islands, Sunda Shelf margin, (9) Myanmar, (10) Philippines and Sulawesi, (11) Great Barrier Reef, (12) Solomon Islands, (13) Fiji, (14) Samoa, (15) Hawaii, (16) Hawaii and Polynesia, (17) Polynesia, (18) Pitcairn, (19) Gulf of California and Easter Island, (20) Mexico, (21) Panama, Colombia, Ecuador. The map was generated in ArcMap 10.3.1 (https://www.esri.de/support-de/produkte/arcgis-for-desktop-10-3) and modified in Adobe Photoshop CS6 (http://www.adobe.com/de/products/catalog.html). Data was processed in R (https://cran.r-project.org/).

The maps derived from the Minimum Convex Polygon (MCP) overlay and the Species Distribution Model (SDM) overlay show largely congruent main richness patterns by revealing the Central Indo-Pacific, and particularly the central Philippines as the center of species richness in symbiont-bearing larger benthic foraminifera (LBF; Fig. 2a,b). Out of the 68 LBF species identified for the entire Indo-Pacific realm, the maximum number for a region is 56 species for the central Philippines in both analyses.

### Minimum Convex Polygon (MCP) modeling

According to the MCP-derived richness map, more than 51 and up to 56 species ranges overlap in the Philippine Archipelago including the Visayas, southern Luzon, northern Mindanao, and large parts of the Sulu Sea (Fig. 2a). More than 46 species ranges overlap in the area around this core (i.e. northern Luzon, southern Mindanao, large parts of Palawan) including parts of north-eastern Indonesia (i.e. Sulawesi, Moluccas, West-Irian Jaya). Ranges of more than 41 species overlap in Palau, southern Indonesia, and the northern Great Barrier Reef. More than 36 species overlap in the Ryukyu Islands (Japan), the South China Sea, Borneo, large parts of southern and eastern Indonesia, southern Papua, the Great Barrier Reef, and the western coast of New Caledonia.

Species richness decreases gradually with distance from the center in both longitudinal and latitudinal direction. At the western margins of the Indian Ocean, the ranges of up to 21 species overlap in the Red Sea and the Persian Gulf, the northern half of the Mozambique Channel, and along the coast of India. Towards the eastern margins of the Pacific Ocean, the ranges of up to 21 species overlap in Hawaii and in the waters around the northern Tuamotu Islands. The distribution ranges of a maximum of six species overlap at the coasts and around the islands of the Eastern Tropical Pacific from southern California down to the Galapagos Islands.

### Species Distribution Modeling (SDM)

The Principal Component Analysis (PCA) transformed the set of original environmental variables obtained from Bio-ORACLE into different sets for modeling application in MaxEnt. PC1 is slightly positively correlated with sea surface temperature variables (*sstmin, sstmean*) and the inversely related importance of diffuse attenuation and chlorophyll a concentration (*damax, damean, damin, chlomax, chlomean, chlomin*), indicated by their highly negative contribution (Table S4). Both, chlorophyll a concentration and dissolved attenuation are indicators for trophic levels. PC2 is strongly positively correlated with sea surface temperature variables (*sstmean, sstmin*) and maximum cloud cover (*cloudmax*), and strongly negatively correlated with dissolved oxygen (*dissox*) and maximum photosynthetic available radiation (*parmax*). PC3 is slightly positively correlated with sea surface temperature variables (*sstmax, sstmean, sstmin*) and strongly positively correlated with mean photosynthetic available radiation (*parmean*). PC4 and PC5 are strongly positively correlated with nutrient variables (*nitrate, phos, silicate*), and PC5 is mostly driven by a slightly positive correlation with the range of chlorophyll a concentration (*chlorange*). The evaluation of the variable contribution implies that for most of the species PC4 had the highest explanatory power (Fig. S2), meaning that nutrient concentration was deemed the most useful parameter, followed by temperature (mostly controlling PC2). The first six principle components explain 83.64% of the variance (Table S4). PC1 explains 29.3% of the variation, PC2 explains 22.7%, and PC3 explains 13.5% (cumulative 65.55%). The remaining PCs 4, 5, and 6 together explain 18.09%.

The average performance of the MaxEnt models is considered significantly better than random (mean AUC_test_ = 0.843; median AUC_test_ = 0.856; range AUC_test_ = 0.549–0.976; Table S5; Fig. S1). Maximum species richness is indicated for the Philippine Central Visayas region, more precisely the Visayas Sea, Guimaras Strait, Gulf of Panay and smaller adjacent localities within and around the coastlines of the Sulu Sea. Here, occurrence probability is given for 52 to 56 species. Between 47 and 51 species are suggested to find favorable conditions in large parts of the Visayas and the islands and coasts of the Sulu Sea, as well as around the islands of Palau, reef areas in southern Sulawesi, the Flores and Banda Sea, and smaller areas in the Great Barrier Reef. Habitat suitability for 42 to 46 species is suggested for large seascapes in the Philippine region (including the northern coast of Borneo), large parts of the coasts of Sulawesi and the northern coast of Java, parts of West-Irian Jaya and the northern coast of Australia (Arufa Sea), the Torres Strait, the Great Barrier Reef, and southern Papua (amongst others).

As seen in the longitudinal gradient (Fig. 2d), the highest peak in richness around 120–125° corresponds to the Philippines, eastern and southern Indonesia, and the Timor Sea. Highest richness is generally confined to the boundaries of the Coral Triangle. A second peak at around 145° corresponds from the Great Barrier Reef to the northern Marianas. Isolated areas of high richness were also detected beyond the longitudinal boundaries of the Coral Triangle (e.g. Myanmar). High Indian Ocean species richness values are suggested for the Maldives, Mascarenes, Seychelles, Mozambique and within the westernmost areas around the Mozambique Channel and the Red Sea. Around the Chagos Archipelago, in contrast to the Maldives in the north, areas suggested for species richness are extremely restricted. With generally around 20 species and up to 32 species in smaller, isolated areas richness for the Mozambique Channel is predicted higher than for the Red Sea where the number is generally 14–18, with single spots reaching 23 species. The Persian Gulf shows less favorable conditions with predicted suitability for around 10 to 15 species in the northern part.

From the center of richness longitudinally towards the East, richness declines more sharply. The Hawaiian Islands are generally predicted suitable for 7–10 species of LBF, with a few exceptions (20–21 species). At around −140° a final peak occurs around the Tuamotus (max. 20 species) in the tropical central Southern Pacific. Further to the eastern Pacific margin, species richness/suitability declines sharply and remains low (<6). It drops significantly within the longitudinal range of the Eastern Pacific Barrier, an area that stretches diagonal south-eastwards from Hawaii through the Pacific. Longitudinal maximum values (<20) of richness at the eastern Pacific margin refer to very restricted and isolated areas of a few kilometers.

The latitudinal gradient of Indo-Pacific LBF richness shows a broad unimodal and asymmetric pattern with the highest peak around 10° North corresponding to the center of richness in the Philippines (Fig. 2c). Almost all maximum richness values are confined to the latitudinal range of the Coral Triangle (CT). From between 15° to 20° North and South, there is a sharp decline in latitudinal richness. The decline in species richness is slightly steeper towards the North than to the South.

## Discussion

The species richness suggested by the Species Distribution Models (SDMs) correlates well with the stacked Minimum Convex Polygon (MCP) pattern, and generally well with empirically observed records of regional LBF species richness. This first LBF species-level modeling provides strong support for previous observations assigning the Central Indo-Pacific as the center of tropical marine biodiversity^11,43^. LBF species richness decreases from its center in latitudinal and longitudinal directions. The decrease towards higher latitudes and towards the eastern margins of the oceans is a general pattern in tropical marine biodiversity^44^. The asymmetric pattern of the LBF latitudinal richness gradient is in agreement with previous analyses on single and overall latitudinal marine richness gradients^45^. It has been identified to be mainly driven by temperature and is most sharply delineated by the extent of the 20 °C winter isotherm^13,46^. Our findings of a sharp decline between 15 to 20° North and South support the notion that the 20° winter isotherm has a strong effect on the richness pattern in LBF. The longitudinal gradient, in turn, very well reflects the dependence on available shallow water habitat. Peaks in richness correlate directly with the position of islands, shallow seas, and continental shorelines. The extreme low at around −120° reveals the impact of deep water expanses (i.e. Eastern Pacific Barrier). Both land-barriers and deep water expanses have been previously identified as the most effective boundaries for tropical marine shelf biotas^46^.

In both of the models, richness is highest in tropical and subtropical waters in the Indian and Pacific Ocean. Additionally, this evaluation is the first to identify a defined geographic region of maximum species-richness in LBF, namely the central Philippine archipelago. Our analyses show the highest species richness scores and the largest extent of most suitable area within the Philippine Visayas region (56 species max.). The area where most distribution ranges overlap appears to also offer the most suitable environmental conditions for LBF. The main pattern agrees with findings reported on overall tropical marine biodiversity^12^ and especially with patterns of shore fish and invertebrates^8,11,12^^,^. The area of maximum richness identified for habitat-forming taxa (i.e. corals, seagrasses, mangroves), in turn, is somewhat extended and includes large parts of southern and eastern Indonesia^12^. So far, the highest number of coral species was recorded in the Sulu Sea ecoregion and comprises Palawan, parts of Borneo, and parts of Mindanao^47^. However, as research is ongoing and several ecoregions are under revision, these scores are subject to change^7^.

Although the Philippines might seem well-sampled (Fig. 1), most of the local occurrence data included in our analysis derive from circumnavigating ship cruises with limited numbers of LBF species recorded from the majority of the sampling locations (see List S1). Recent sampling on shallow (max. 30 m) nearshore reefs in northern Palawan, however, revealed that regional LBF species richness is among the highest (39 species; unpubl. data, Table S2) ever reported so far.

Contrary to the MCP, the SDM projection allows a distinction of habitat suitability over the full geographic space including unsampled areas, and thus identifies coldspots and hotspots of potential LBF species richness within the center of biodiversity. According to the SDM, larger areas with high scores outside the Philippines are also indicated for central and eastern Indonesia, the northernmost coast of Australia, southern Papua, the Great Barrier Reef and the Torres Strait.

Unevenly distributed species diversity across taxa within the center of biodiversity is related to strong dependency on habitat heterogeneity, e.g. cross-shelf gradients in salinity, turbidity, water energy levels and substrate types^6,12,43^. Areas with a deep photic zone and moderate hydrodynamic energy are most likely to exhibit high numbers of LBF species, as this promotes the occurrence of species that are specialized to narrow ranges of light intensities along the depth gradient^23,24^. Species diversity is further known to be linked to available area/habitats^25^. Larger areas usually offer higher spatial diversity (i.e. habitat heterogeneity), and also allow species to have larger spatial ranges and a larger population size. This, in turn, reduces the risk of extinction and promotes vicariance. Available nearshore habitat (i.e. coastline length), was recently identified as the best predictor for species richness and overall biodiversity in the Central Indo-Pacific, followed by habitat heterogeneity and sea surface temperature^12^. Most available nearshore habitat is found in the central Philippines and in eastern Indonesia, as both areas harbor several hundreds of smaller islands and islets that offer a great variety of habitats and resources^12^. Additionally, the geological history of the Philippines is highly complicated^48^ and may have contributed significantly to the diversification of the region^8^. It is assumed that island integration events during the Miocene and Pliocene promoted bioconcentration and an amalgamation of separately evolved faunas^8^. Vicariance events^49^ like the Pleistocene isolation of sea basins^50^ might have further stimulated speciation, especially within the central Philippines^8^. Today, the Indo-West Pacific, the Great Barrier Reef, and the tropical western Indian Ocean are characterized by a high level of connectivity and were identified acting as a source for larval dispersal^51^.

The decrease of tropical marine biodiversity towards the coasts of the Eastern Pacific is steeper than towards the western margins of the Indian Ocean, especially beyond the Central Pacific^13,44^. Only a few”transpacific” species of reef organisms are found both in the central Indo-Pacific and in the Eastern Tropical Pacific (ETP)^52^. Our analysis reveals the same general pattern. Among all 68 LBF species analyzed in this study, at least eight have a transpacific distribution with occurrences in the ETP (Table S2). They represent 12% of all LBF species identified herein. A further species (*Dendritina*? *culebraensis* (McCulloch)) is probably endemic to the ETP (Table S2).

The reef fauna of the ETP went largely extinct after the built-up of the Panama Isthmus during the Pliocene and, to date, remains depauperate^44^. Prevalent environmental constraints are the limitation of available shallow water habitats, the eastward decrease of sea surface temperatures, the impact of the cold-water Peru and California currents, the lowering of the thermocline, the presence of upwelling zones at the eastern margins of the Pacific, and the isolation by the Eastern Pacific Barrier (EPB)^19,44^.

The EPB is a broad and deep stretch of open ocean lacking stepping stone islands or atolls facilitating the dispersal of warm water biotas across biogeographic boundaries. It has a long geological record and has persisted throughout the Cenozoic^53^. To cross this most efficient marine biogeographic barrier, organisms are required to possess long-lived pelagic larvae or propagules that tolerate temporary exposure to low sea surface temperatures^19,54^. Based on individual survival capabilities, foraminiferan propagules settle and survive in a cryptic state at least for weeks to months in environments that are potentially unfavorable for adult specimens^55^. Adult forms, in turn, are dispersed passively by ocean currents, by attaching to floating objects or migrating organisms, by anthropogenic vectors, or within the fecal pellets of herbivorous fish^13,56,57^. Oceanic currents function as vectors for dispersal, or as physical barriers for passive dispersing of marine biotas where the directionality of the prevailing current impedes faunal connectivity and gene flow^51,56^. Although El Nino events may potentially facilitate eastward directed range extensions of tropical species^52,53^, eastward dispersal across the EPB and a replenishment by western population sources towards the ETP are considered highly unlikely^58^. This suggests that the ETP LBF biotas have been largely isolated from central Pacific propagule supplies.

The overall richness pattern in the Indian Ocean reveals a fundamental difference from that in the Pacific Ocean as tropical marine biodiversity increases towards the western margin of the Indian Ocean (Madagascar, Mozambique Channel, Red Sea)^7,59^. Our SDMs provides strong support for this observation (Fig. 2b). The coastlines of the northern Mozambique Channel represent the biodiversity center for the Indian Ocean what appears to be driven by the directionality of the South Equatorial Current (SEC) that ensures high connectivity from E to W^51,59^. Central Indian Ocean island chains (e.g. Chagos-Laccadive Ridge), that represent transitional zones between the central Indo-Pacific and the western Indian Ocean biotas^60^, may facilitate dispersal and range expansion over large distances and potentially act as stepping stones^59,60^. However, in contrast to the Maldives that have a generally rich benthic foraminiferal fauna^61^ and are characterized by the presence of several central Indo-Pacific LBF taxa^13^, the LBF fauna of the Chagos Archipelago is relatively depauperate^62^. The low LBF diversity is reflected by the islands isolated position^62^ and the restricted richness shown in our SDM projection. In the western Indian Ocean province that includes the Chagos Archipelago^60^, characteristic central Indo-Pacific LBF taxa (e.g. *Marginopora*, *Cycloclypeus*, *Alveolinella*, *Calcarina*) are absent and partially substituted by morphologically similar species (e.g. *Cycloclypeus carpenteri* by *Heterocyclina tuberculosa*)^13^.

Within the northern Mozambique Channel, the SDM projection suggests suitable environmental conditions for up to 32 species. Actual species records reveal a range overlap of 21 species in this area with maximum records of about 14 species for individual sites (Kenia and Zanzibar; Table S2). Potential distribution ranges of LBF, projected via SDM, were previously shown to be not fully utilized^35^ but possibly identify priority sites for future colonization. Because previous analyses indicate that the observed LBF diversity matches on average 35% of the true diversity^19^ additional sampling activities may be required. For the Red Sea, the second most diverse region of the Indian Ocean^59^, the number of LBF species predicted to occur was consistent with the total number of species recorded so far (20 species).

As suggested by the SDM results, the pattern of high biodiversity at the margins of the western Indian Ocean is interrupted by a highly unsuitable zone around the Gulf of Aden and the Somali coast (Fig. 2b). This area represents one of the main seasonal upwelling areas of the world with local reefs known to be less diverse and less developed^59^. Despite the overlapping of about 18 LBF species as seen in the MCP and recently reported occurrences of 12 species from the southern Yemeni coast^63^, LBF richness is suggested to be extremely low. Similar outputs are known from SDM projections for scleractinian corals that show relative restricted^64^ or no suitability at all^65^.

Previous evaluations of LBF biogeography on generic level are largely consistent with our results (Fig. 1). Langer & Hottinger^13^ examined the distribution of 19 LBF genera of which 16 were monospecific. They identified an inner, high diversity Central Indo-Pacific biogeographic province ranging from Okinawa to the Sahul Shelf that is largely characterized by the restricted occurrence of various calcarinid taxa. As illustrated by Langer in Goldbeck^66^, LBF diversity peaks somewhere within the center of that province with a maximum number of 27 genera. In discussing the global shifts in marine biodiversity over time, Renema *et al*.^26^ presented a recent hotspot zone stretching from the South China Sea down to the Sahul Shelf and Fiji. Previous analyses of Belasky^19^ revealed a high correlation of scleractinian coral and LBF diversity patterns and suggested a generic-level LBF hotspot within an area stretching from Borneo to New Guinea but excluding most of the Philippines.

Sea surface temperatures and the trophic level have been identified as the main constraints on LBF distribution^13,19,35^. The distribution ranges of recent LBF taxa are restricted by the 14–20 °C minimum winter isotherms with varying individual tolerances towards lower temperatures^13^.

Our SDM analysis of factors regulating the distribution of each species supports the importance of oligotrophic conditions and elevated sea surface temperatures (Table S4; Fig. S2). Several species, most of them calcarinids, revealed an especially high dependence on warm-water conditions (Table S5). The Calcarinidae are unique elements of the Indo-Pacific foraminiferal reef biotas. They are extremely abundant on reef crests in the Western Tropical Pacific, vital producers of calcium carbonate and are deposited as extensive beach sands^13,17,67^. Calcarinidae, with the exception of the small *Neorotalia calcar*, exhibit the most restricted distribution ranges and are known for their comparatively narrow tolerance towards lower temperatures^13,35^ and elevated nutrient levels^68^.

An increase in species richness towards the Central Indo-Pacific is observed in all six LBF families. With 21 species present in the entire Indian and Pacific Ocean, we identified the Peneroplidae as by far the species-richest family and the Alveolinidae as the least diverse (3 species).

The geographic coverage on the distribution of recent Indo-Pacific LBF has steadily improved since the mid to late 1990s. The delineation of biogeographic patterns, however, still constitutes a challenge as LBF distribution is often patchy and species-level taxonomy requires further efforts. Most of the studies included herein focused on shallow water depths within the limit of recreational diving (<50 m). Fewer studies included grab-sampled stations at greater depth and deeper dwelling species of LBF generally occur more sporadically in the literature. Future developments in LBF biogeography, including continuous efforts to resolve molecular species identifications, are likely to improve the resolution of the observed distribution ranges and taxonomic relationships. However, the species richness pattern identified herein is strongly supported by its remarkable similarity to those of other tropical marine taxa in revealing the Philippines as the bullseye of tropical marine biodiversity.

In view of today’s rapid global warming, the outlook for tropical reef calcifiers is alarming^69–71^. This study contributes to the completion of the overall picture of tropical marine biodiversity and the knowledge of richness patterns may aid our understanding to target conservation actions^72^.

## Electronic supplementary material

Supplementary Information

Supplementary Table S2
