# Supplementary material for: Patterns of species richness and the center of diversity in modern Indo-Pacific larger foraminifera
Source: Sci Rep. 2018 May 29;8:8189. doi: 10.1038/s41598-018-26598-9 (PMC5974165; doi:10.1038/s41598-018-26598-9)
Supplement: Supplementary file 1 — Supplementary Information [file 41598_2018_26598_MOESM1_ESM.pdf]

Supplementary Information

Patterns of species richness and the center of diversity in modern Indo-Pacific larger foraminifera

Meena Förderer*[1], Dennis Rödder[2], Martin R. Langer*[1]

[1]Steinmann Institute for Geology, Mineralogy and Paleontology, Department of Earth Sciences, University of Bonn, Nussallee 8, 53115 Bonn, Germany
[2]Herpetology Section, Zoological Research Museum Alexander Koenig, Adenauerallee 160, 53113 Bonn, Germany

*SI correspondence to:
Martin R. Langer, martin.langer@uni-bonn.de
Meena Förderer, meena.foerderer@gmail.com

Supplementary Figure S1: Test AUC range. AUC values range between 0.5487 (minimum; outlier) and 0.9762 (maximum). Mean AUC is 0.843, median AUC is 0.856. Figure generated in Past3.

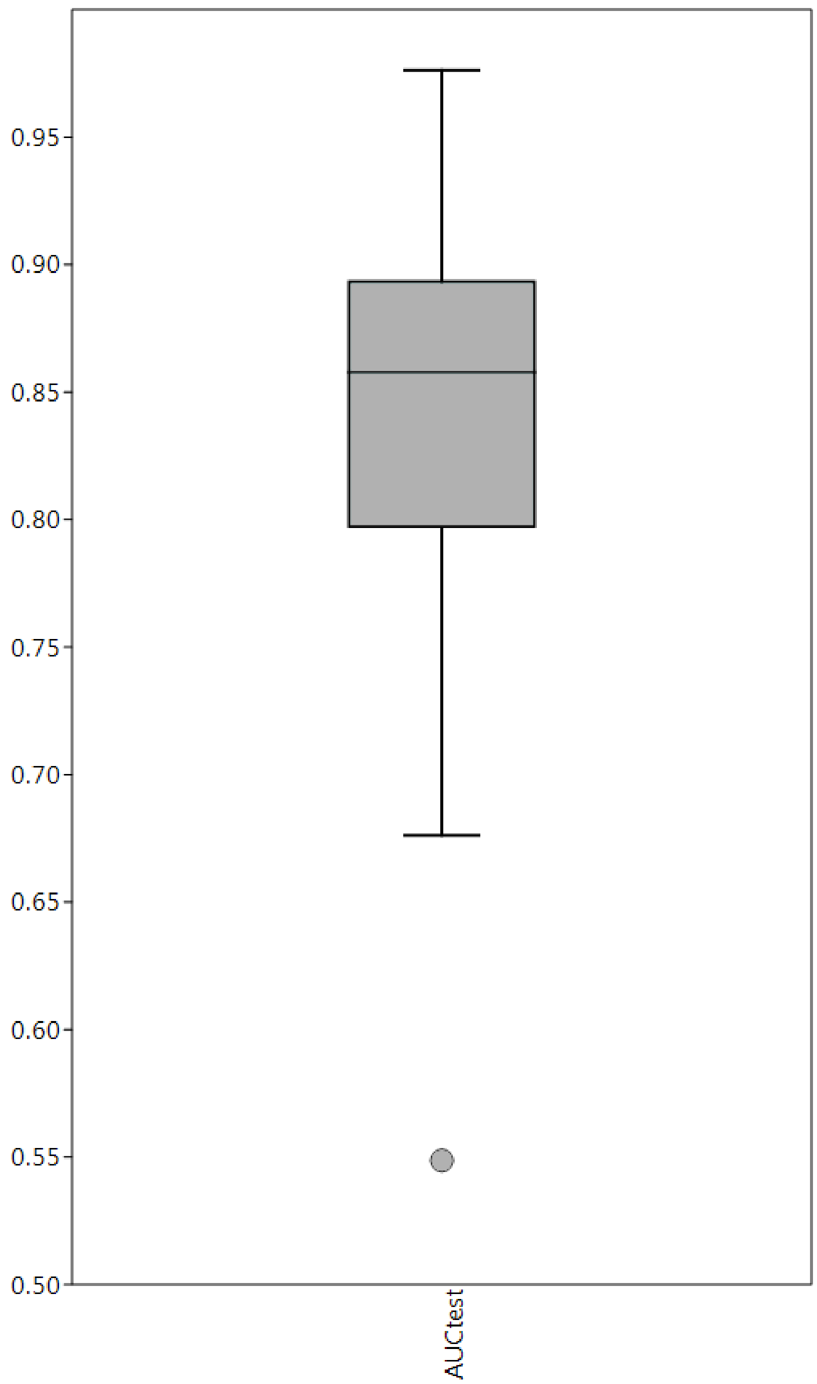

Supplementary Figure S2: Principle Components (PCs) contributions. Comparison of the contributions of the six PCs to the species distributions indicates that PC4 reveals the highest influence for most species, followed by PC2 and PC1. PC6 is the least important. Figure generated in Past3.

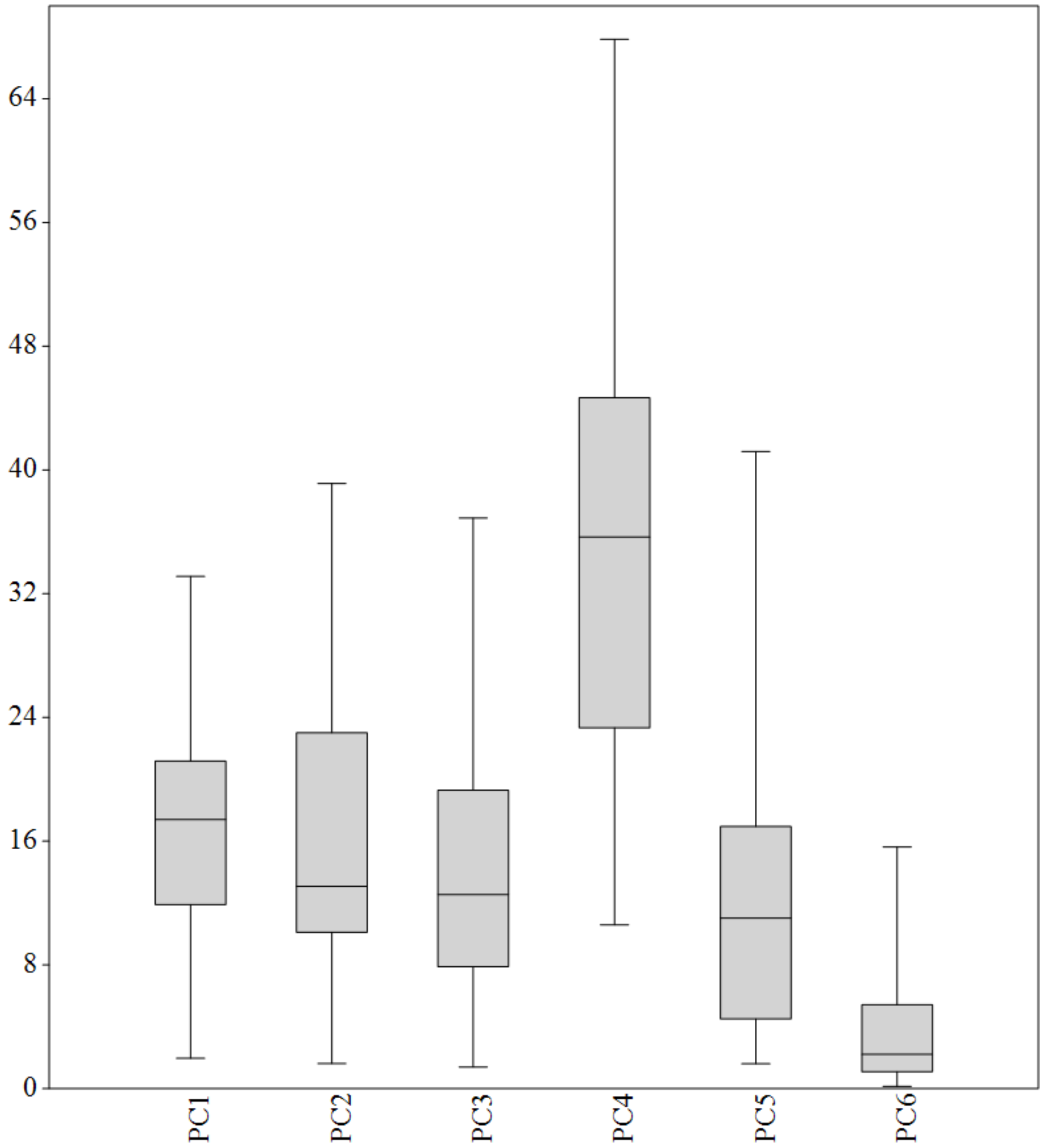

Supplementary List S1: Studies included for establishing the richness maps. The 114 literature records are numbered and correspond to the references in Tbl. S5.

1) **Abu-Zied RH, Bantan RA. 2013.** Hypersaline benthic foraminifera from the Shuaiba Lagoon, eastern Red Sea, Saudi Arabia: Their environmental controls and usefulness in sea-level reconstruction. *Marine Micropaleontology* 103:51-67.
2) **Albani AD. 1968.** Recent Foraminiferida from Port Hacking, New South Wales. *Contributions from the Cushman Laboratory for Foraminiferal Research* 19:85-119.
3) **Baccaert J. 1987.** Distribution patterns an taxonomy of benthic foraminifera in the Lizard Island Reef Complex, northern Great Barrier Reef, Australia. Ph.D. thesis, Université de Liège.
4) **Bandy OL, Arnal RE. 1957.** Distribution of recent foraminifera off west coast of Central America. *Bulletin of the American Association of Petroleum Geologists* 41:2037-2053.
5) **Betjeman KJ. 1969.** Recent foraminifera from the western continental shelf of Western Australia. *Contributions from the Cushman Foundation for Foraminiferal Research* 20:119-138.
6) **Bhalla SN. 1970.** Foraminifera from Mariana Beach sands, Madras, and faunal provinces of the Indian Ocean. *Contributions from the Cushman Foundation for Foraminiferal Research* 21:156-163.
7) **Bicchi E, Debenay JP, Pagès J. 2002.** Relationship between benthic foraminiferal assemblages and environmental factors in atoll lagoons of the central Tuamotu Archipelago (French Polynesia). *Coral Reefs* 21:275-290.
8) **Brady HB. 1884.** Report on the foraminifera dredged by H. M. S. Challenger during the years 1873-1876. *Reports of the Scientific Results of the Voyage of H. M. S. Challenger, Zoology* 9:1-814.
9) **Cann JH, Clarke JDA. 1993.** The significance of *Marginopora vertebralis* (Foraminifera) in the surficial sediments at Esperance, Western Australia, and in last interglacial sediments in northern Spencer Gulf, South Australia. *Marine Geology* 111:171-187.
10) **Chapman F. 1901.** Foraminifera from the lagoon at Funafuti. *Journal of the Linnaean Society of London, Zoology* 28: 161-210.
11) **Chapman F, Parr WJ. 1938.** Australian and New Zealand species of the foraminiferal genera *Operculina* and *Operculinella*. *Proceedings of the Royal Society of Victoria* 50:279-299.
12) **Cheng TC, Zheng SY. 1978.** The recent foraminifera of the Xisha Islands, Guangdong Province, China, I. *Studia Marina Sinica* 12:149-227. [Chinese with summary and new genera and species in English]
13) **Cherif OH, Al-Ghadban AN, Al-Rifaiy IA. 1997.** Distribution of foraminifera in the Arabian Gulf. *Micropaleontology* 43:253-280.
14) **Chiji M, Lopez SM. 1968.** Regional foraminiferal assemblages in Tanabe Bay, Kii Peninsula, Central Japan. *Publications of the Seto Marine Biological Laboratory* 16:85-125.
15) **Church CC. 1968.** Shallow water foraminifera from Cape San Lucas, Lower California. *Proceedings of the California Academy of Sciences* 30:357-380.
16) **Clearly DFR, Renema W. 2007.** Relating species traits of foraminifera to environmental variables in the Spermonde Archipelago, Indonesia. *Marine Ecology Progress Series* 334:73-82.
17) **Collen JD, Garton DW. 2004.** Larger foraminifera and sedimentation around Fongafale Island, Funafuti Atoll, Tuvalu. *Coral Reefs* 23:445-454.
18) **Culver SJ, Mallinson DJ, Corbett DR, Leorri E, Rouf AA, Shazili NAM, Yaacob R, Whittaker JE, Buzas MA, Parham PR. 2012.** Distribution of foraminifera in the Setiu estuary and lagoon, Terengganu, Malaysia. *Journal of Foraminiferal Research* 42:109-133.
19) **Cushman JA. 1914.** A monograph of the foraminifera of the North Pacific Ocean, part 4. Chilostomellidae, Globigerinidae, Nummulitidae. *Bulletin of the United States National Museum* 71:1-46.
20) **Cushman JA. 1915.** A monograph of the foraminifera of the North Pacific Ocean, part 5. Rotaliidae. *Bulletin of the United States National Museum* 71:1-81.
21) **Cushman JA. 1917.** A monograph of the foraminifera of the North Pacific Ocean, part 6. Miliolidae. *United States National Museum Bulletin* 71:1-108.
22) **Cushman JA. 1921.** Foraminifera of the Philippine and adjacent seas. *Smithsonian Institution, United States National Museum Bulletin* 100:1-608.
23) **Cushman JA. 1924.** Samoan Foraminifera. *Publications of the Carnegie Institution of Washington*, no. 342, *Departement of Marine Biology* 21:1-85.
24) **Cushman JA. 1933.** The foraminifera of the tropical pacific collections of the “Albatross”, 1899-1900, part 2. Lagenidae to Alveolinellidae. *Smithsonian Institution United States National Museum Bulletin* 161:1-79.
25) **Cushman JA, Todd R, Post RJ. 1954.** Recent foraminifera from the Marshall Islands, Bikini Atoll and nearby atolls, Part 2, Oceanography (biologic). *Professional Papers, U.S. Geological Survey* 260H: 319-384.
26) **Dawson JL, Smithers SG, Hua Q. 2014.** The importance of large benthic foraminifera to reef island sediment budget and dynamics at Raine Island, northern Great Barrier Reef. *Geomorphology* 222:68-81.

Supplementary List S1 continued.

27) **Debenay JP. 2012.** *A Guide to 1,000 Foraminifera from Southwestern Pacific, New Caledonia*. Paris: Editions IRD Marseille/Publications Scientifiques du Muséum.
28) **Fajemila OT, Langer MR, Lipps JH. 2015.** Spatial patterns in the distribution, diversity and abundance of benthic foraminifera around Moorea (Society Archipelago, French Polynesia). PloS ONE 10(12): e0145752.
29) **Fornasini C. 1908.** Illustrazione di specie orbignyane di Nodosaridi, di Rotalidi e d'altri foraminiferi. *Memorie della Reale Accademia della Scienze del l' Istituto di Bologna, Scienze Naturali* 6:41-54.
30) **Fujita K. 2004.** A field colonization experiment on small-scale distributions of algal symbiont-bearing larger foraminifera on reef rubble. *Journal of Foraminiferal Research* 34:169-179.
31) **Gabrié C, Montaggioni L. 1982.** Sediments from fringing reefs of Réunion Island, Indian Ocean. *Sedimentary Geology* 31:281-301.
32) **Graham JJ, Militante PJ. 1959.** Recent Foraminifera from the Puerto Galera Area Northern Mindoro, Philippines. *Stanford University Publications, Geological Sciences* 6:1-171.
33) **Gudmundsson G. 1994.** Phyologeny, ontogeny and systematics of recent Soritacea Ehrenberg 1839 (Foraminiferida). *Micropaleontology* 40:101-155.
34) **Haig DW. 1988a.** Miliolid Foraminifera from the inner neritic sand and mud facies of the Papuan Lagoon, New Guinea. *Journal of Foraminiferal Research* 18: 203-236.
35) **Haig DW. 1988b.** Distribution of miliolid foraminifera in marine sediments around Motupore Island, Papua New Guinea. *Science in New Guinea* 14: 54-94.
36) **Haig DW. 1997.** Foraminifera from Exmouth Gulf, Western Australia. *Journal of the Royal Society of Western Australia* 80:263-280.
37) **Hallock PM. 1977.** Some aspects of the ecology of several large, symbiont-bearing foraminifera and their contribution to warm, shallow-water biofacies. Ph.D. thesis, Biological Oceanography, University of Hawaii.
38) **Hallock P. 1984.** Distribution of selected species of living algal symbiont-bearing foraminifera on two pacific coral reefs. *Journal of Foraminiferal Research* 14:250-261.
39) **Hatta A, Ujiié H. 1992a.** Benthic foraminifera from Coral Sea between Ishigaki and Iriomote Islands, Southern Ryukyu Island Arc, Northwest Pacific, Part 1, systematic descriptions of Textulariina and Miliolina. *Bulletin of the College of Science, University of Ryukyus* 53:49-119.
40) **Hatta A, Ujiié H. 1992b.** Benthic foraminifera from Coral Sea between Ishigaki and Iriomote Islands, Southern Ryukyu Island Arc, Northwest Pacific, Part 2, systematic descriptions of Rotaliina. *Bulletin of the College of Science, University of Ryukyus* 54:163-287.
41) **Haunold TG, Baal C, Piller WE. 1997.** Benthic foraminiferal associations in the Northern Bay of Safaga, Red Sea, Egypt. *Marine Micropaleontology* 29:185-210.
42) **Hayward BW, Grenfell H, Reid CM, Hayward KA. 1999.** Recent New Zealand shallow-water benthic foraminifera: taxonomy, ecologic distribution, biogeography, and use in paleoenvironmental assessment. *Institute of Geological and Nuclear Sciences Monograph* 21:1-258.
43) **Heron-Allen E, Earland A. 1915.** The foraminifera of the Kerimba Archipelago (Portugese East Africa), part II. *Transactions of the Zoological Society of London* 20:543-794.
44) **Hofker J. 1927.** The Foraminifera of the Siboga Expedition. Part 1. Families Tinoporidae, Rotaliidae, Nummulitidae, Amphisteginidae. *Siboga Expeditie, Monographie Iva.* Leiden. E.J. Brill:1-78.
45) **Hofker J. 1930.** The Foraminifera of the Siboga Expedition. Part 2.Families Astorhizidae, Rhizamminidae, Reophacidae, Anomalinidae, Peneroplidae. *Siboga Expeditie, Monographie Iva.* Leiden. E.J. Brill: 79-170.
46) **Hofker J. 1933.** *Foraminifera of the Malay Archipelago*. Papers from Dr. Th. Mortensen's Pacific Expedition, 1914-16, Pacific Expedition. Videnskabelige meddelelser fra Dansk Naturhistorisk Forening i København, 93. Leiden, Netherlands.
47) **Hofker J. 1950.** Recent Peneroplidae Part 1. *Journal of the Royal Microscopical Society* 70:388-396.
48) **Hofker J. 1968.** Foraminifera from the Bay of Jakarta, Java. *Bijdragen tot de Dierkunde Afl.* 37:11-59.
49) **Hofker J. 1978.** Biological results of the Snellius Expedition XXX. The foraminifera collected in 1929 and 1930 in the Eastern Part of the Indonesian Archipelago. *Zoologische Verhandelingen, Rijksmuseum von Natuurlijke Historie te Leiden* 161:1-69.
50) **Hohenegger J. 1994.** Distribution of living larger Foraminifera NW of Sesoko-Jima, Okinawa, Japan. PSZNI *Marine Ecology* 15:291-334.
51) **Hohenegger J. 1996.** Remarks on the distribution of larger foraminifera (Protozoa) from Belau (Western Carolines). *Kagoshima University Research Center for the Pacific Islands, Occasional Papers* 30:85-90.
52) **Hohenegger J, Yordanova E, Nakano Y, Tatzreiter F. 1999.** Habitats of larger foraminifera on the upper reef slope of Sesoko Island, Okinawa, Japan. *Marine Micropaleontology* 26: 109-168.
53) **Holzmann M, Hohenegger J, Hallock P, Piller WE, Pawlowski J. 2001.** Molecular phylogeny of large miliolid foraminifera (Soritacea Ehrenberg 1839). *Marine Micropaleontology* 43:57-74.

Supplementary List S1 continued.

54) **Hottinger L. 1980.** Répartition comparée des grands Foraminifères de la Mer Rouge et de l'Ocean Indien. *Annali Università Ferrara (Nova Serie) Sezione* 4:1-13.
55) **Hottinger L, Halicz E, Reiss Z. 1993.** *Recent Foraminiferida from the Gulf of Aqaba, Red Sea.* Slovenska Akademija Znanosti in Umetnosti, Ljubljana, Classis IV, Historia Naturalis, Opera 3, 1-179.
56) **Hughes GW. 1977.** Recent from the Honiara Bay Area, Solomon Islands. *Journal of Foraminiferal Research* 7:45-47.
57) **Hughes GW. 1985.** Recent foraminifera and selected biometrics of Heterostegina depressa from Otong Java Atoll, Solomon Islands, Southwest Pacific. *Journal of Foraminiferal Research* 15:13-17.
58) **Hughes GW. 1995.** Recent foraminifera from inter-reef channels, nearshore North Rarotonga, Cook Islands, South Pacific. *Journal of Micropaleontology* 14:29-36.
59) **Kuwano Y. 1956.** Invertebrate fauna of the intertidal zone of the Tokara Islands, XII Foraminifera. *Publications of the Seto Marine Biological Laboratory* 2:141-150.
60) **Langer MR, Lipps JH. 2003.** Foraminiferal distribution and diversity, Madang Reef and Lagoon, Papua New Guinea. *Coral Reefs* 22:143-154.
61) **Langer MR, Lipps JH. 2006.** Assembly and persistence of foraminifera in introduced mangroves on Moorea, French Polynesia. *Micropaleontology* 52:343-355.
62) **Langer MR, Makled WA, Pietsch SJ, Weinmann A. 2009.** Asynchronous calcification in juvenile megalospheres: An ontogenetic window into the life cycle and polymorphism of *Peneroplis. Journal of Foraminiferal Research* 39:8-14.
63) **Langer MR, Thissen JM, Makled WA, Weinmann AE. 2013b.** The foraminifera from the Bazaruto Archipelago (Mozambique). *N. Jb. Geol. Paläont. Abh.* 267:155-170.
64) **Le Calvez Y. 1965.** Les foraminifères; p. 181-201 in: Guilcher, A., Berthois, L., Le Calvez, Y., Battistini, R., Crosnier, A., *Les recifs coralliens et le lagon de l'ile Mayotte (Archipel des Comores, Oceans Indien)*. Memoire Office de la Recherche Scientifique et Technique Outre-Mer.
65) **Lee JJ, Burnham B, Cevasco ME. 2004.** A new modern soritid foraminifer, Amphisorus saurensis n. sp., from the Lizard Island Group (Great Barrier Reef, Australia). *Journal of Micropaleontology* 50:357-368.
66) **Lessard RH. 1980.** Distribution patterns of intertidal and shallow-water foraminifera of the tropical pacific ocean. *Cushman Foundation Special Publication* 19:40-58.
67) **Levy A, Mathieu R, Poignant A, Rosset-Moulinier M, Ambroise D. 1996.** Foraminifères benthiques des iles Maldives (océan Indien). *Memoires de la Société Géologiques de France* 169:129-138.
68) **Lobegeier MK. 2002.** Benthic foraminifera of the family Calcarinidae from Green Island Reef, Great Barrier Reef province. *Journal of Foraminiferal Research* 32:201-216.
69) **Loeblich AR, Tappan H. 1994.** Foraminifera of the Sahul Shelf and Timor Sea. *Cushman Foundation for Foraminiferal Research,* Special Publication 31:1-661.
70) **Makled WA, Langer MR. 2011.** Benthic Foraminifera from the Chuuk Lagoon Atoll System (Caroline Islands, Pacific Ocean). *N. Jb. Geol. Paläont. Abh.* 259:231-249.
71) **McCulloch I. 1977.** Qualitative Observations on Recent Foraminiferal Tests with Emphasis on the Eastern Pacific: Parts I-III. *Los Angeles: University of Southern California*, vi + 1079.
72) **McKee ED, Chronic J, Leopold EB. 1959.** Sedimentary belts in lagoon of Kapingamaringi Atoll. *Bulletin of the American Association of Petroleum Geologists* 43:501-562.
73) **Möbius KA. 1880.** Foraminifera von Mauritius; p. 65-112 in Möbius, K., Richters, F. & von Martens, E., *Beiträge zur Meeresfauna der Insel Mauritius und der Seychellen.* Berlin: Gutman.
74) **Murray JW. 1965.** The Foraminiferida of the Persian Gulf. Part 2. The Abu Dhabi Region. *Paleogeography, Paleoclimatology, Paleoecology* 1:307-332.
75) **Murray JW. 1966a.** The Foraminiferida of the Persian Gulf. Part 3. The Halat al Bahrani Region. *Paleogeography, Paleoclimatology, Paleoecology* 2:59-68.
76) **Murray JW. 1966b.** The Foraminiferida of the Persian Gulf. Part 4. Khor al Bazam. *Paleogeography, Paleoclimatology, Paleoecology* 2:153-169.
77) **Murray JW. 1970a.** The Foraminiferida of the Persian Gulf. Part 6. Living forms in the Abu Dhabi Region. *Journal of Natural History* 4:55-67.
78) **Murray JW. 1970b.** The foraminifera of the hypersaline Abu Dhabi Lagoon, Persian Gulf. *Lethaia* 3:51-68.
79) **Murray JW. 1994.** Larger foraminifera from the Chagos Archipelago: their significance for Indian Ocean biogeography. *Marine Micropaleontology* 24:43-55.
80) **Narayan YR, Pandolfi JM. 2010.** Benthic foraminiferal assemblages from Moreton Bay, South-East Queensland, Australia: Applications in monitoring water and substrate quality in subtropical estuarine environments. *Marine Pollution Bulletin* 60:2062-2078.

Supplementary List S1 continued.

81) **Nobes K, Uthicke S. 2008.** Benthic foraminifera of the Great Barrier Reef. A guide to species potentially useful as Water Quality Indicators: Australian Institute of Marine Science, Townsville. Report to the Marine and Tropical Sciences Research Facility. Reef and Rainforest Research Centre Limited, Cairns, p44 available at: http://www.rrrcorgau/publications/downloads/371-AIMS-Nobes-etal-2008-Benthic-Foraminifera-of-the-GBR.pdf
82) **Oki K. 1989.** Ecological analysis of benthonic foraminifera in Kagoshima Bay, South Kyushu, Japan. South Pacific Study, Kagoshima University Research Center for the South Pacific 10:1-191.
83) **Orbigny Ad'. 1826.** Tableau méthodique de la classe des Céphalopodes. *Annales des Sciences Naturelles* 7:245-314.
84) **Parker J. 2009.** Taxonomy of Foraminifera from Ningaloo Reef, Western Australia. *Memoirs of the Association of Australasian Paleontologists* 36:1-810.
85) **Parker J, Gischler E. 2011.** Modern foraminiferal distribution and diversity in two atolls from the Maledives, Indian Ocean. *Marine Micropaleontology* 78:30-49.
86) **Parker JH, Gischler E. 2015.** Modern and relict foraminiferal biofacies from a carbonate ramp, offshore Kuwait, northwest Persian Gulf. *Facies* 61:10.
87) **Parker WK, Jones TR. 1860.** On the nomenclature of the foraminifera. 4 (continued). *Annales and Magazine of Natural History*, series 3, 4:333-351.
88) **Pignatti J, Frezza V, Benedetti A, Carbone F, Accordi G, Matteucci R. 2012.** Recent foraminiferal assemblages from mixed carbonate-siliciclastic sediments of southern Somalia and eastern Kenya. *Bollettino della Società Geologica Italiana* 131:47–65.
89) **Renema W. 2002.** Larger foraminifera as marine environmental indicators. *Scripta Geologica* 124:1-263.
90) **Renema W. 2003.** Larger foraminifera on reefs around Bali (Indonesia). *Zoologische Verhandelingen Leiden* 345:337-366.
91) **Renema W. 2006a.** Large benthic foraminifera from the deep photic zone of a mixed siliciclastic-carbonate shelf off East Kalimantan, Indonesia. *Marine Micropaleontology* 58:73-82.
92) **Renema W. 2006b.** Habitat variables determining the occurrence of large benthic foraminifera in the Berau area (East Kalimantan, Indonesia). *Coral Reefs* 25:351-359.
93) **Renema W. 2008.** Habitat selective factors influencing the distribution of larger benthic foraminiferal assemblages over the Kepulauan Seribu. *Marine Micropaleontology* 68:286-298.
94) **Renema W. 2009.** Is increased calcarinid (foraminifera) abundance indicating a larger role for macro-algae in Indonesian Plio-Pleistocene coral reefs?. *Coral Reefs* 29:165-173.
95) **Renema W, Hoeksema BW, van Hinte JE. 2001.** Larger benthic foraminifera and their distribution patterns on the Spermonde shelf, South Sulawesi. *Zoologische Verhandelingen Leiden* 334:115-149.
96) **Renema W, Hohenegger J. 2005.** On the identity of *Calcarina spengleri* (Gmelin, 1791). *Journal of Foraminiferal Research* 35:15-21.
97) **Rhumbler L. 1906.** Foraminiferen von Laysan und den Chatham Inseln. *Zoologischer Jahresbericht* 24:21-80.
98) **Smith RK. 1968.** An intertidal Marginopora colony in Suva Harbor, Fidji. *Contributions from the Cushman Foundation for Foraminiferal Research* 19:12-17.
99) **Smith R. 1995.** Sand and aggregate resources Majuro Atoll, Marshall Islands. South Pacific Applied Geoscience Commission, Technical Report 215:57 p.
100) **Thissen JM, Langer MR. 2017.** Spatial patterns and structural composition of foraminiferal assemblages from the Zanzibar Archipelago (Tanzania). *Palaeontographica*, Abt. A: Palaeozoology – Stratigraphy Article Vol. 308, Issues 1–3:1–67.
101) **Todd R. 1957.** Geology of Saipan, Mariana Islands, Part 3. Paleontology. Smaller foraminifera. *Professional Papers U.S. Geological Survey* 280H:265-320.
102) **Todd R. 1961.** Foraminifera from the Onotoa Atoll Gilbert Islands. *United States Geological Survey Professional Paper* 354H:171-191.
103) **Todd R. 1965.** The foraminifera of the tropical pacific collections of the "Albatross", 1899-1900, Part 4. Rotaliform families and planctonic families. *Smithsonian Institution United States National Museum Bulletin 161*, v + 139.
104) **Van Marle LJ.1988.** Bathymetric distribution of benthic foraminifera on the Australian-Irian Jaya continental margin, eastern Indonesia. *Marine Micropaleontology* 13:97-152.
105) **Vénec-Peyré MT, Salvat B. 1981.** Les Foraminifères de l'Atoll de Scilly (Archipel de la Société): Etude comparée de la biocénose et da la thanatocénose. *Annales Institute Océanographie* 57:79-110.
106) **Whittaker JE, Hodgkinson RL. 1995.** The Foraminifera of the Pitcairn Islands. In: Benton, T.G. & Spencer, T., The Pitcairn Islands: Biogeography, ecology and prehistory. *Biological Journal of the Linnean Society* 56:365-371.
107) **Yasukochi T, Kayanne H, Yamaguchi T, Yamano H. 2014.** Sedimentary facies and Holocene depositional processes of Laura Island, Majuro Atoll. *Geomorphology* 222:59-67.

Supplementary List S1 continued.

108) **Yordanova EK, Hohenegger J. 2002.** Taphonomy of Larger Foraminifera: Relationships between Living Individuals and Empty Tests on Flat Reef Slopes (Sesoko Island, Japan). *Facies* 46:169-2004.

109) **Yordanova EK, Hohenegger J. 2004.** Morphoclines of living operculinid foraminifera based on quantitative characters. *Journal of Micropaleontology* 50:149-177.

110) **Yuvaraja M, Ramanujam N. 2013.** Occurance of cross breeding of benthic foraminifera in South Andaman India. *International Journal of Current Research* 5:681-683.

111) **Yuvaraj M, Ramanujam N, Siva Sankar R. 2013.** Distribution of benthic foraminifera in South Andaman, India. *International Journal of Current Research* 5:562-566.

112) **Zapata JM, Olivares JM. 2000.** Biodiversidad y zoogeografía de los foraminiferos bentonicos de Isla de Pascua (27°10’ S, 109°20’ W), Chile. Boletin de la Sociedad de Biologia de Conception (Chile) 71:53-77.

113) **Zheng SY. 1979.** The Recent foraminifera of the Xisha Islands, Guangdong Province, China. II. *Studia Marina Sinica* 15:101-232.

114) **Zheng SY. 1980.** The Recent foraminifera of the Zhonsha Islands, Guangdong Province, China. I. *Studia Marina Sinica* 16:143-182.

Supplementary Table S1: Species of LBF identified for the Indo-Pacific. List of all symbiont-bearing larger benthic foraminiferal species recorded for the Indo-Pacific and sorted alphabetically within their families.

Family	Species	Reference for identification	Distribution range from literature	Remarks
**ALVEOLINIDAE** Ehrenberg, 1839	***Alveolinella quoyi*** (d'Orbigny, 1826)	Parker, 2009, p. 83, fig. 59a-g	Longitudinally from the Maldives (***54, 67, 85*** ; 72° E) to Hawaii (***8*** ; 157° W), latitudinally from Tanabe Bay, Japan (***50*** , ***52*** , ***108*** ; 26° N) to Queensland, Australia (***81*** ; 27° S). It is absent from the coasts and islands of the Tropcial Eastern Pacific and the western Indian Ocean.	*A. quoyi* has a restricted Indo-Pacific distribution. It prefers open to shallow back-reef habitats and is highly adapted to oligotrophic conditions (Murray 1991). The species is at time assigned to the fossil *A. boscii* (Defrance).
	Borelis pulchra (d'Orbigny, 1839a)	Cheng & Zheng, 1978, p. 202, pl. 18, fig. 1a-c	Longitudinally from Zanzibar (***100*** ; 39° E) to Socorro Island, Mexico (***71*** ; 111° W), latitudinally from Hawaii (***66*** ; 22° N) to the Pitcairn Islands (***106*** ; 24° S).	*B. pulchra* is originally described from the Atlantic. The species is sometimes assigned to the Miocene *B. melo* (Fichtel & Moll).
	Borelis schlumbergeri (Reichel, 1937)	Hottinger *et al* ., 1993, p. 68, pl. 75, figs 1-17	Longitudinally from the Red Sea (***41*** ; 33° E) to Clarion Island, Mexico (***71*** ; 114° W), latitudinally from the Gulf of Aqaba (***55*** ; 29° N) to the Exmouth Gulf, western Australia (***36*** ; 22° S).	*B. schlumbergeri* is also reported from the Atlantik. It is the only species of *Borelis* in the Red Sea and along the eastern coast of Africa (Reiss & Hottinger 1984).
**PENEROPLIDAE** Schultze, 1854	***Coscinospira hemprichii*** Ehrenberg, 1839	Hottinger *et al* ., 1993, p. 69, pl. 76, figs 1-12; pl. 77, figs 1-8	Longitudinally from Bazaruto, Mozambique (***63*** ; 35° E) to the Gulf of California (***71*** ; 110° W), latitudinally from the Gulf of Aqaba (***55*** ; 29° N) to New Caledonia (***27*** ; 22° S).	This species is often confounded with *Peneroplis arietinus* (Batsch; see list of synonymizations Tbl. S2).
	Dendritina ambigua (Fichtel & Moll, 1798)	Hohenegger 2011, figs on p. 43, 44	Longitudinally from Bombay, India (***71*** ; 72° E) to Saipan, Marianas (***101*** ; 142° E), latitudinally from the Ryukyu Islands (***50, 51*** ; 26° N) to the Exmouth Gulf, western Australia (***36*** ; 22° S).	At times confounded with *Dendritina striata* Hofker. Azazi (1990) reports *D. ambigua* from the northern Red Sea. Despite the fact, that this species is originally reported from the Red Sea by Fichtel & Moll (1798), the pictures given by Azazi do not allow a profound synonymization. Therefore the outermost record towards the East African coast identified within this study is on the western coast of India (synonymized species of McCulloch 1977).
	Dendritina ? culebraensis (McCulloch, 1977)	McCulloch, 1977, p. 230, pl. 100, fig. 12	One record from the Eastern Tropical Pacific, Costa Rica (***71*** ; 85° W, 10° N).	
	Dendritina striata Hofker, 1951	Hofker, 1951, p. 234, text figs 12-14; Hatta & Ujiié, 1992a, p. 78, pl. 15, figs 5a, b (not figs 4a, b, 6a, b)	Longitudinally from western Java (***93*** ; 106° E) to Palmyra, Line Islands (***material examined*** ; 162° W), latitudinally from the Ryukyu Islands (***39*** ; 24° N) to western Java (***93*** ; 5° S).	At times confounded with *Dendritina ambigua* (Fichtel & Moll).
	Dendritina striatopunctata Hofker, 1951	Hofker, 1951, p. 233, text figs 10, 11	Longitudinally from western Java (***48*** , ***49*** ; 106° E) to the Great Barrier Reef (***3*** ; 145° E), latitudinally from the Ryukyu Islands (***39*** ; 24° N) to Ningaloo Reef, western Australia (***84*** ; 21° S).	
	Dendritina waikikiensis McCulloch, 1977	McCulloch, 1977, p. 230, pl. 100, fig. 18	One record from the eastern Indo-Pacific, Hawaii (***71*** ; 157° W, 21° N).	
	Dendritina zhengae Ujiié *in* Hatta & Ujiié, 1992	Cheng & Zheng, 1978, p. 194, 259, pl. 15, figs 9-12 [not *Dendritina pacifica* McCulloch, 1977]; Hatta & Ujiié, 1992a, p. 78, pl. 15, figs 2a-3b	Longitudinally from the Xisha Islands, South China Sea (***12*** ; 111° E) to the Marshall Islands (***25*** ; 166° E), latitudinally from the Ryukyu Islands (***52*** , ***108*** ; 26° N) to the Spermonde Shelf (***89*** , ***95*** ; 4° S).	
	Dendritina* cf. *D. zhengae Ujiié *in* Hatta & Ujiié	Hohenegger, 2011, figs on p. 43, 44	Longitudinally from the Gulf of Aden (***47*** ; 43° E) to the Ryukyu Islands (***50*** , ***52*** , ***108*** ; 127° E), latitudinally from the Ryukyu Islands (***50*** , ***52*** , ***108*** ; 26° N) to western Java (***47*** ; 5° S).	
	Euthymonacha polita (Chapman, 1900)	Parker, 2009, p. 103, figs 72a-h, 73a-j	Longitudinally from Bazaruto, Mozambique (***63*** ; 35° E) to the Tuamotu Islands (***24*** ; 143° W), latitudinally from the Ryukyu Islands (***39*** ; 24° N) to Ningaloo Reef, western Australia (***84*** ; 23° S). It is absent from Hawaii and the coasts of the Tropcial Eastern Pacific.	
	Laevipeneroplis bradyi (Cushman, 1930)	Cushman, 1930, p. 40, pl. 14, figs 8-10; Baccaert, 1987, not *Sorites discoideus* (Flint), p. 63, pl. 21, figs 2, 3; pl. 22; figs 1, 2; pl. 23, fig. 1; pl. 24, fig. 1	From two localities in the central Indo-Pacific. One from Raja Ampat (***material examined*** ; 130° E, 0° S) and the other from Lizard Island, Great Barrier Reef (***3*** ; 145° E, 14° S).	
	Laevipeneroplis malayensis (Hofker, 1952)	Renema, 2002, p. 94, pl. 1, figs e, f [not *Laevipeneroplis proteus* (d'Orbigny)]	Longitudinally from western Java (***93*** ; 106° E) to the Johnston Atoll (***66*** ; 169° W), latitudinally from the Johnston Atoll and the Xisha Islands (***66*** , ***12*** ; 16° N) to the Timor Sea (***104*** ; 9° S).	This speciesis often confounded with the Atlantic *Laevipeneroplis proteus* (d'Orbigny) and *Archaias angulatus* (Fichtel & Moll; synonymization list in Tbl. S2). It is uncertain if *L. proteus* and *L. malayensis* are the same species. Further study is required to reveal their relationship.
	Monalysidium acicularis (Batsch, 1791)	Parker, 2009, p. 138, figs 98a-h, 99a-e	Longitudinally from the Gulf of Aqaba (***55*** ; 34° E) to the Tuamotu Islands (***24*** ; 143° W), latitudinally from the Gulf of Aqaba (***55*** ; 29° N) to New South Wales, Australia (***2*** ; 34° S). It is absent from the coasts of the Eastern Tropical Pacific.	
	Monalysidium confusa (McCulloch, 1977)	McCulloch, 1977, p. 231, pl. 100, fig. 9; Debenay, 2012, p. 111, 281	From two locations, the southern Philippines (***71*** ; 121° E, 6° N) and New Caledonia (***27*** ; 166°E, 22° S).	
	Monalysidium dissimilis (McCulloch, 1977)	McCulloch, 1977, p. 231, pl. 100, fig. 14	Two records, one from northern Palawan, Philippines (***material examined*** ; 119° E, 11° N) and the other from the central Visayas (***71*** ; 122° E, 10° N).	
	Monalysidium limatula (McCulloch, 1977)	McCulloch, 1977, p. 232, pl. 100, fig. 6	One record is available from the southern Philippines (***71*** ; 121° E, 6° N).	
	Monalysidium okinawaensis (Hatta & Ujiié, 199	Parker, 2009, p. 141, figs 100a-k, 101a-g, 102a-k	Longitudinally from Ningaloo Reef, western Australia (***84*** ; 113° E) to Raja Ampat (***material examined*** ; 130° E), latitudinally from the Ryukyu Islands (***40*** ; 24° N) to the Ningaloo Reef (***84*** ; 23° S).	
	Peneroplis antillarum d'Orbigny, 1839a	Gudmundsson 1994, Britsh Solomon Isles and Japan, p. 111, text figs 19, 20; pl. 3, fig. 4; pl. 4, fig. 4; Hohenegger 2011, p. 43, figs on p. 39, 44	Longitudinally from Palawan, Philippines (***material examined*** ; 119° E) to the Solomon Islands (***57*** ; 159° E), latitudinally from the Ryukyu Islands (***52*** , ***108*** ; 26° N) to Queensland, Australia (***80*** ; 27° S).	*P. antillarum* is originally described from the Atlantic and reported as rare by d'Orbigny (1839a; Gudmundsson 1994). It is very often confounded with *P. planatus* (Fichtel & Moll) and *P. pertusus* (Forskal). A helpful description and analysis of this species has been made by Gudmundsson (1994).

Supplementary Table S1 continued.

Family	Species	Reference for identification	Distribution range from literature	Remarks
**PENEROPLIDAE** Schultze, 1854	***Peneroplis arietinus*** (Batsch, 1791)	Baccaert, 1987, p. 60, pl. 19, figs 3a-b; pl. 20, figs 1-4; pl. 21, figs 1a, b	Longitudinally from the Andaman Islands (***110*** ; 92° E) to the Gulf of California (***15*** ; 109° W), latitudinally from the Ryukyu Islands (***39*** , ***66*** ; 24° N) to Queensland, Australia (***80*** ; 27° S).	*P. arietinus* is often confounded with either *P. pertusus* (Forskal) or *Coscinospira hemprichii* Ehrenberg.
	Peneroplis pertusus (Forskal, 1775)	Hohenegger, 2011, p. 27, 43, figs on p. 43, 45; Gudmundsson, 1994, p. 115, text figs 23, 24; pl. 3, figs 1, 3	Longitudinally from Zanzibar (***100*** ; 39° E) to the Pacific coast of Panama (***4*** ; 81° W), latitudinally from the Tokara Islands (***59*** ; 29° N) to the Easter Island (***111*** ; 27° S).	*P. pertusus* is commonly confounded with *P. planatus* (Fichtel & Moll), *P. arietinus* (Batsch) and *Coscinospira hemprichii* Ehrenberg.
	Peneroplis planatus (Fichtel & Moll, 1798)	Cimerman & Langer, 1991, p. 50, pl. 50, figs 1-6	Longitudinally from the Red Sea (***41*** ; 33° E) to the Easter Island (***111*** ; 109° W), latitudinally from the Gulf of Aqaba (***55*** ; 29° N) to Queensland, Australia (***2*** ; 34° S).	*P. planatus* (Fichtel & Moll) is commonly confounded with *P. pertusus* (Forskal) and *P. antillarum* d'Orbigny.
	***Peneroplis* ? sp.**	Langer *et al* ., 2009, p. 10, pl. 1, figs 1-13, 16-18	Two records from the central Indo-Pacific, one from Palawan, Philippines (***material examined*** ; 119° E, 11° N) and the other from the Caroline Islands (***62*** ; 151° E, 7° N).	
**SORITIDAE** Ehrenberg, 1839	***Amphisorus hemprichii*** Ehrenberg, 1839	Hottinger *et al* ., 1993, p. 71, pl. 81, figs 1-8; pl. 82, figs 1-11	Longitudinally from the Gulf of Aqaba (***55*** ; 34° E) to Cocos Island, Costa Rica (***71*** ; 86° W), latitudinally from the Tokara Islands (***59*** ; 29° N) to southwestern Australia (***9*** ; 33° S).	*Amphisorus hemprichii* is commonly confounded with *Sorites orbiculus* (Forskal) and *Marginopora vertebralis* Quoyi & Gaimard. Thicker tests and additional rows of apertures appear in shallower environments (Hohenegger pers. comment, June 2013).
	Amphisorus kudakajimaensis (Gudmundsson, 1994)	Gudmundsson, 1994, p. 128, text figs45-48; pl. 6, figs 1, 2; pl. 7, fig. 4; pl. 8, fig. 2	Longitudinally from the Ryukyu Islands (***33*** ; 127° E) to Hawaii (***33*** ; 157° W), latitudinally from the Ryukyu Islands (***33*** ; 26° N) to Palau (***51*** ; 7° N).	*A. kudakajimaensis* might probably be documented from more locations. Gudmundsson (1994) mentioned, that part of the specimens illustrated by Hofker 1930 from the central Indo-Pacifics Siboga Expedition that were labeled as *Marginopora vertebralis* (pl. LXII, figs 1, 5) are in fact *A. kudakajimaensis* . However, Hofker did not mention the exact location of these specimens that he identified as the "A1 form". Gudmundsson further noted that specimen slides from the modern faunas of Bikini and Rongerik labeled as *M. vertebralis* are in fact *M. kudakajimaensis* specimens. Carpenter (1883, pl. 6, fig. 5) mentioned *Orbitolites* specimens from the Challenger Expedition which he labeled as "sub-typical" specimens. For assignment of these specimens, the original material needs to be examined.
	Amphisorus sauronensis Lee, Burnham & Cevasco, 2004	Lee *et al* ., 2004, p. 366, text figs 3-15, 17	Longitudinally from Ningaloo Reef, western Australia (***84*** ; 113° E) to New Caledonia (***27*** ; 166° E), latitudinally from Borneo (***92*** ; 2° N) to New Caledonia (***27*** ; 22° S).	
	Cyclorbiculina compressa (d'Orbigny, 1839)	Loeblich & Tappan, 1994, p. 62, pl. 111, figs 1-4	One record from the Timor Sea (***69*** ; 128° E, 10° S).	It was previously assumed, that this species only occurs in the Atlantic (Langer & Hottinger, 2000). Further study is required to clarify the taxonomic relationships between the populations from the Atlantic and Indo-Pacific.
	Marginopora vertebralis Quoy & Gaimard, in Blainville 1830	Debenay, 2012, p. 109, 282	Longitudinally from La Réunion (***31*** ; 55° E) to Easter Island (***106*** ; 128° W), latitudinally from the Tokara Islands (***59*** ; 29° N) to western Australia (***5*** ; 28° S).	Hottinger (1980) noted that the distribution limit of *Marginopora* in the Indian Ocean lies west of the Seychelles and east of Mauritius.
	Parasorites orbitolitoides (Hofker, 1930)	Debenay, 2012, p. 113, 282; Lehmann, 1961, p. 645, text figs 34, 38; pl. 10, figs 1-5	Longitudinally from Bazaruto, Mozambique (***63*** ; 35° E) to Easter Island (***111*** ; 109° W), latitudinally from the Ryukyu Islands (***50*** , ***52*** , ***108*** ; 26° N) to Easter Island (***111*** ; 27° S).	*Parasorites orbitolitoides* is often confounded with *Sorites marginalis* (Lamarck), which is very similar especially in the juvenile stage. Lehmann (1961) provides a very helpful comparison of the two species. The taxonomic relationship to the similar *Sorites variabilis* (Lacroix) from the Red Sea needs to be clarified.
	Sorites marginalis (Lamarck, 1816)	Lehmann, 1961, p. 643, text figs 31-33, 35, 37; pl. 8, figs 9, 10; pl. 9, figs 1-6	Longitudinally from the Quirimbas (***material examined*** ; 40° E) to the Tuamotus (***24*** ; 142° W), latitudinally from Hawaii (***38*** ; 21° N) to New Caledonia (***27*** ; 22° S).	*S. marginalis* is often confounded with either *S.orbiculus* (Forskal) or *Parasorites orbitolitoides* (Hofker), which is very similar especially in the juvenile stage. Lehmann (1961) provides a very helpful comparison and analysis of the Soritidae. The taxonomic relationship to the similar *Sorites variabilis* (Lacroix) from the Red Sea needs to be clarified.
	Sorites orbiculus (Forskal, 1775)	Hottinger *et al* ., 1993, p. 72, pl. 83, figs 1-13; Lehmann, 1961, p. 641, text fig. 36, pl. 8, figs 1-8	Longitudinally from the Red Sea (***41*** ; 33° E) to the Pitcairn Islands (***106*** ; 128° W) and latitudinally from the Gulf of Aqaba (***55*** ; 29° N) to Queensland, Australia (***2*** ; 34° S).	*S. orbiculus* is often confounded with either *S. marginalis* (Lamarck) or *Amphisorus hemprichii* Ehrenberg. Lehmann (1961) provides a very helpful comparison and analysis of the Soritidae.
	Sorites variabilis Lacroix, 1941	Hottinger *et al* ., 1993, p. 73, pl. 84, figs 1-15	From the Gulf of Aqaba (***54*** , ***55*** ; 34° E, 27° and 29° N).	Very similar to *Parasorites orbitolitoides* (Hofker). The taxonomic relationship needs to be clarified.
**AMPHISTEGINIDAE** Cushman, 1927a	***Amphistegina bicirculata*** Larsen, 1976	Hottinger *et al* ., 1993, p. 132, pl. 182, figs 1-11; pl. 183, figs 1-7	Longitudinally from the Red Sea (***41*** ; 33° E) to the Easter Island (***111*** ; 109° W), latitudinally from southern Japan and the Red Sea (***55, 59*** ; 29° N) to Queensland, Australia and the Easter Island (***80, 111*** ; 29° S).	
	Amphistegina lessonii d'Orbigny, 1826	Hottinger *et al* ., 1993, p. 132, pl. 184, figs 1-11; pl. 185, figs 1-7	Longitudinally from the Red Sea (***41*** ; 33° E) to Hawaii (***37, 38*** ; 157° W), latitudinally from the Gulf of Aqaba (***55*** ; 29° N) to New Caledonia (***27*** ; 22° S).	
	Amphistegina lobifera Larsen, 1976	Hottinger *et al* ., 1993, p. 133, pl. 186, figs 1-10; pl. 187, figs 1-7; pl. 188, figs 1-6	Longitudinally from the Red Sea (***41*** ; 33° E) to the Pitcairn Islands (***106*** ; 128° W), latitudinally from the Gulf of Aqaba (***55*** ; 29° N) to the Pitcairn Islands (***106*** ; 25° S).	

Supplementary Table S1 continued.

Family	Species	Reference for identification	Distribution range from literature	Remarks
**AMPHISTEGINIDAE** Cushman, 1927a	***Amphistegina madagascariensis*** d'Orbigny, 1826	McCulloch, 1977, p. 410, pl. 154, figs 8, 9	Longitudinally from Madagascar (***83*** ; 49° E) to the Tuamotus (***103*** ; 138° W), latitudinally from Hawaii (***71*** ; 21° N) to the Tuamotus (***103*** ; 19° S).	*A. madagascariensis* is resembling *A. lessonii* d'Orbigny. The species probably represents a variety of the latter, however, as very characteristic specimens have been found in our sample material from Raja Ampat (Indonesia) and Palawan (Philippines) we consider it to be a distinct species. The original record of d'Orbigny is from Madagascar, however, as no precise location was available the chosen reference point is estimated according to availability of suitable shallow water habitat.
	Amphistegina papillosa Said, 1949	Hottinger *et al* ., 1993, p. 134, pl. 189, figs 1-10; pl. 190, figs 1-7	Longitudinally from the Gulf of Aqaba (***55*** ; 34° E) to the Bikini Atoll (***25*** ; 165° E), latitudinally from the Gulf of Aqaba (***55*** ; 29° N) to Ningaloo Reef, western Australia (***84*** ; 23° S).	
	Amphistegina radiata (Fichtel & Moll, 1798)	Debenay, 2012, p. 216, 319	Longitudinally from the Gulf of Aqaba (***55*** ; 34° E) to Rarotonga (***58*** ; 159° W), latitudinally from southern Japan (***14*** ; 33° N) to Queensland, Australia (***80*** ; 27° S).	
**CALCARINIDAE** d'Orbigny, 1826	***Baculogypsina sphaerulata*** (Parker & Jones, 1860)	Hottinger & Leutenegger, 1980, p. 125, pl. 9	Longitudinally from Bali (***90*** ; 115° E) to Alofi, Niue (***103*** ; 169° W), latitudinally from the Tokara Islands (***59*** ; 29° N) to New Caledonia (***27*** ; 22° S).	
	Baculogypsinoides spinosus (Yabe & Hanzawa, 1930)	Hohenegger, 2011, p. 34, 42, 60	Longitudinally from eastern Java (***46*** , ***48*** , ***49*** , ***93*** ; 106° E) to New Caledonia (***27*** ; 166° W), latitudinally from the Ryukyu Islands (***50*** , ***52*** , ***108*** ; 26° N) to New Caledonia (***27*** ; 22° S).	
	Calcarina calcarinoides Cheng & Zheng, 1978	Zheng, 1979, p. 219, 260, pl. 25, figs 8a-c, 9a, b, 10-12; pl. 26, figs 1a-c, 2-11; pl. 32, fig. 10	One record is available from the Xisha Islands, South China Sea (***12***) at Longitude 111° E and Latitude 16° N.	
	Calcarina defrancei d'Orbigny, 1826	Hottinger & Leutenegger 1980, p. 124, pls 2, 3, 7: 4-6	Longitudinally from the Maldives (***67*** , ***85*** ; 72° E) to the Pacific Island Alofi (***103*** ; 169° W), latitudinally from the Tokara Islands (***59*** ; 29° N) to the southern Great Barrier Reef (***81*** ; 29° S).	At times confounded with *Calcarina spengleri* and *Neorotalia calcar* (see listed synonymizations in Tbl. S2). The spines of specimens occurring outside and at the margins of the central Indo-Pacific realm (e.g. Maldives, Great Barrier Reef) appear to be shorter and less pronounced.
	Calcarina exuberans Debenay, 2012 new name for *Calcarina hispida* Brady var. *pulchella* Chapman 1900	Debenay 2012, p. 188, 323	From the southern Central Indo-Pacific at Funafuti (***10*** ; 179° E, 8° S) and New Caledonia (***27*** ; 166° E, 22° S).	
	Calcarina gaudichaudii d'Orbigny *in* Ehrenberg 1840	Hottinger & Leutenegger, 1980, p. 124, pls 4, 5	Longitudinally from Borneo (***92*** ; 118° E) to the Marshall Islands (***107*** ; 171° E), latitudinally from the Ryukyu Islands (***59*** ; 26° N) to Raja Ampat (***material examined*** ; 0° S).	At times confounded with *Calcarina spengleri* (see listed synonymizations in Tbl. S2).
	Calcarina hispida Brady, 1876	Renema & Hohenegger 2005, p. 18, pl. 1, figs 1-13 (not figs 14-19)	Latitudinally from southern Japan (***14*** ; 33° N) to the southern Great Barrier Reef (***81*** ; 23° S).	Often confounded with *Calcarina mayori* (see listed synonymizations in Tbl. S2).
	Calcarina luzonensis McCulloch 1977	McCulloch 1977, p. 432, pl. 155, fig. 7	One record from eastern Luzon, Philippines (***71*** ; 123° E, 13° N).	
	Calcarina mayori Cushman, 1924	Cushman 1924, p. 44, pl. 14, figs 4-7; Renema & Hohenegger 2005, p. 16, fig. 2, pl. 1, figs 14-21 (not figs 22-24)	Longitudinally from north off Jakarta (***93*** ; 106° E) to Samoa (***8*** ; 170° E), latitudinally from the Ryukyu Islands (***52*** ; 26° N) to the southern Great Barrier Reef (***81*** ; 23° S).	*Calcarina mayori* is often confounded with *C. hispida* and *C. spengleri* (see listed synonymizations in Tbl. S2).
	Calcarina quoyi d'Orbigny, 1826	Hohenegger 2011, p. 56, figs on p. 58	Longitudinally from the east coast of Borneo (***92*** ; 118° E) to the Mariana Islands (***29*** ; 145° E), latitudinally from the Ryukyu Islands (***50*** ; 26° N) to Sulawesi (***92*** ; 4° S).	*C. quoyi* d'Orbigny was widely considered a *nomen nudum* and a synonym of *C. hispida* Brady (e.g. Fornasini 1908; Hatta & Ujiié 1992b; Cushman *et al* . 1954; Cushman 1921), however, other authors more recently identified it as a species distinct from *C. hispida* (Hohenegger 2011; Renema 2006b). The specimens of d'Orbigny depicted by Fornasini (1908) probably show two different species. In the original description, *C. quoyi* is reported from the Marianas (without giving an explicit location) and from "Rawack" (most probably the island "Rauki" north off Waigeo, Raja Ampat (Indonesia)). However, the latter location was not included in the analyses as no species of *C. quoyi* were found in our material from Raja Ampat.
	Calcarina spengleri (Gmelin, 1791)	Renema & Hohenegger 2005, p. 16, fig. 1, pl. 2, figs 1-10 (not pl. 1, figs 1-10)	Longitudinally from the Bay of Jakarta (***48*** ; 106° E) to the Marshall Islands (***66*** ; 167° E), latitudinally from the Ryukyu Islands (***40*** , ***66*** ; 24° N) to Sulawesi (***69*** ; 11° S).	Often confounded with *Calcarina mayori* , *C. gaudichaudi* , *C. hispida* and *C. defrancei* (see listed synonymizations in Tbl. S2).
	***Calcarina* sp.**	Debenay 2012, p. 189, 323 (*Calcarina* sp. 1); McCulloch 1977, p. 433, pl. 155, fig. 6 (*Calcarina* cf. *rustica* Todd & Post)	One record from western Luzon, Philippines (***71*** ; 123° E, 13° N) and the other from New Caledonia (***27*** ; 166° E, 22° S).	
	Neorotalia calcar (d'Orbigny, 1826)	Hottinger & Leutenegger, 1980, p. 123, pl. 1	Longitudinally from the Bay of Safaga in the Red Sea (***41*** ; 33° E) to the Cook Islands (***103*** ; 159° W), latitudinally from the Gulf of Aqaba (***55*** ; 27° N) to the Abrolhos Islands of western Australia (***5*** ; 28° S).	
	Schlumbergerella floresiana (Schlumberger, 1896)	Renema, 2003, p. 352, fig. 23	Longitudinally from the Java Sea (***44*** ; 113° E) to Timor (***44*** ; 123° E), latitudinally from Mindanao (***44*** ; 4° N) to Timor (***44*** ; 10° S).	Langer & Hottinger (2000) already stated that *S. floresiana* is severely restricted to the inner central Indo-Pacific. However, at that time data coverage was regarded insufficient to plot occurrences on a map and still is scarce even 16 years later with the only records being added since then are from Bali (Renema 2003).
**NUMMULITIDAE** de Blainville, 1827	***Assilina /Operculina ammonoides*** (Gronovius, 1781)	Hottinger *et al* ., 1993, p. 154, pl. 222, figs 1-8; pl. 223, figs 1-14; pl. 224, figs 1-8; pl. 225, figs 1-9	Longitudinally from the eastern coast of Africa (***material examined*** ; 32° E) to Moorea, French Polynesia (***28*** ; 149° W), latitudinally from Tanabe Bay, Japan (***14*** ; 33° N) to Queensland, Australia (***80*** ; 27° S). It is absent from the coasts and islands of the Eastern Tropical Pacific.	This species is highly morphological variable and has been assigned to other species several times in the literature (see Tbl. S2). It is the most widespread species of *Assilina* /*Operculina* and covers a broad environmental range (Hohenegger *et al* . 2000).

Supplementary Table S1 continued.

Family	Species	Reference for identification	Distribution range from literature	Remarks
**NUMMULITIDAE** de Blainville, 1827	***Assilina*/*Operculina complanata*** (Defrance in Blainville, 1822)	Hohenegger *et al*., 2000, p. 20, pl. 2, figs 13-18	Longitudinally from the Quirimbas (***43***; 40° E) to Tonga (***8***; 175° W), latitudinally from the Persian Gulf (***86***; 28° N) to Tonga (***8***; 20° S).	
	Assilina*/*Operculina discoidalis Cushman, 1921	Hohenegger *et al*., 2000, p. 21, pl. 2, figs 1-6; pl. 5, figs 1-6	Longitudinally from the Zanzibar archipelago (***100***; 39° E) to Fiji (***24***; 179° E), latitudinally from the Ryukyu Islands (***52***; 26° N) to Ningaloo Reef on the western coast of Australia (***84***; 23° S).	
	Assilina*/*Operculina elegans Cushman, 1921	Hohenegger, 2011, p. 64, fig. p. 66	Longitudinally from the western coast of Borneo (***22***; 119° E) to the Ryukyu Islands (***109***; 26° E), latitudinally from the Ryukyu Islands (***109***; 26° N) to Borneo (***22***; 4° S).	The specimens depicted by the authors Cushman (1921), Hohenegger (2011) and McCulloch (1977) are either representing different species or show a high degree of variability of this species. Further study is required to clarify the taxonomic status.
	Assilina*/*Operculina gaimardi d'Orbigny, 1826	Debenay, 2012, p. 228, 326	Longitudinally from the Maldives (***67***; 72° E) to New Caledonia (***27***; 166° E), latitudinally from the Ryukyu Islands (***40***; 24° N) to New Caledonia (***27***; 22° S).	
	Assilina*/*Operculina philippinensis Cushman, 1921	Cushman, 1921, p. 378, text fig. 15	Longitudinally from the South China Sea (***22***; 155° E) to New Caledonia (***27***; 166° E), latitudinally from the South China Sea (***22***; 21° N) to New Caledonia (***27***; 22° S).	Based on the records of Cushman (1921), the species is common in the Philippines and also occurs in marine provinces nearby. Further study is required to clarify the taxonomic status.
	Cycloclypeus carpenteri Brady, 1881	Hohenegger *et al*., 2000, p. 25, pl. 4, fig. 7	Longitudinally from Mayotte (***64***; 45° E) to the Tuamotus (***24***; 143° W), latitudinally from the Ryukyu Islands (***50***; 26° N) to New Caledonia (***27***; 22° S).	*C. carpenteri* is absent from the Red Sea and the East African coast, where the appropriate habitat is occupied by the morphologically very similar *Heterocyclina tuberculata*.
	Heterocyclina tuberculata (Möbius, 1880)	Hottinger *et al*., 1993, p. 156, pl. 226, figs 1-7; pl. 227, figs 1-8; pl. 12, figs 3-7	Two records from the Gulf of Aqaba (***55***; 34° E, 29° and 27° N) and one from Mauritius (***73***; 57° E, 20° S).	The distribution of *H. tuberculata* (Möbius) is restricted to the western Indian Ocean, including the Red Sea (Reiss & Hottinger 1984). Cushman (1921) reports this species from the Philippines, but he gives no figures and the species description is ambiguous. In the Indo-Pacific, the annular *H. tuberculata* is replaced by the morphologically very similar *Cycleoclypeus carpenteri*.
	Heterostegina depressa d'Orbigny, 1826	Hottinger *et al*., 1993, p. 157, pl. 228, figs 1-11; pl. 229, figs 1-8; pl. 230, fig. 9	Longitudinally from the Red Sea (***41***; 33° E) to Ecuador (***71***; 80° W), latitudinally from the Gulf of Aqaba and the Tokara Islands, Japan (***55***, ***59***; 29° N) to Queensland, Australia (***80***; 27° S).	*H. depressa* is the most widespread nummulitid foraminifera. Originally described from the Atlantic Ocean, it is globally distributed in tropical and subtropical marine waters. As most authors nowadays recognize the genus *Heterostegina* as monospecific and represented by the species *H. depressa* (e.g. Hohenegger, Yordanova & Hatta 2000, Langer & Hottinger 2000) *H. curva* Möbius and *H. suborbicularis* d'Orbigny are regarded herein as synonyms (Tbl. S2). However, suspicious specimens that display a great resemblance to Möbius' original description of *H. curva* and the specimens depicted by McCulloch (1979) have been found recently in material from Moorea and Zanzibar. Further study is required to clarify the taxonomy of this genus.
	Nummulites cumingii (Carpenter, 1860)	Hohenegger *et al*., 2000, p. 13, pl. 1, figs 11-18	Longitudinally from the Exmouth Gulf, western Australia (***36***; 114° E) to the Ryukyu Islands (***50***; 127° E), latitudinally from the Ryukyu Islands (***50***; 26° N) to the Exmouth Gulf (***36***; 22° S).	Originally reported from the Philippines by Carpenter (1860), however, without giving a definite location. Commonly confounded with *Nummulites venosus* (Fichtel & Moll). The two species are often regarded as synonymous. A helpful comparison has been made by Hohenegger *et al.* (2000).
	Nummulites venosus (Fichtel & Moll, 1798)	Hohenegger *et al*., 2000, p. 11, pl. 1, figs 1-10; pl. 4, fig. 10	Longitudinally from the Red Sea (***41***; 33° E) to Vava'u, Tonga (***24***; 173° W), latitudinally from the Red Sea (***41***; 26° N) to Shark Bay, Australia (***11***; 25° S).	Commonly confounded with *Nummulites cumingii* (Carpenter). The two species are often regarded as synonymous. A helpful comparison has been made by Hohenegger *et al*. (2000).
	Planoperculina heterosteginoides (Hofker, 1933)	Yordanova & Hohenegger, 2004, p. 152, text figs 2, 13	Longitudinally from western Java (***46***; 105° E) to Tuvalu (***10***; 179° E), latitudinally from the Ryukyu Islands (***109***; 26° N) to the Exmouth Gulf, western Australia (***36***; 22° S).	
	Planostegina longisepta (Zheng, 1979)	Yordanova & Hohenegger, 2004, p. 152, text figs 2, 13	Four records from the central Indo-Pacific, two from the South China Sea (***112*** and ***113***; 114° E, 16° N and 111° E, 16° N) and two from the Ryukyu Islands (***109*** and ***40***; 127° E, 26° N and 124° E and 24° N).	Some authors (e.g. Debenay 2012) regard this species as synonymous with *Planostegina operculinoides* (Hofker).
	Planostegina operculinoides (Hofker, 1927)	Yordanova & Hohenegger, 2004, p. 152, text figs 2, 13	Longitudinally from the Gulf of Aqaba (***55***; 34° E) to New Caledonia (***27***; 166° E), latitudinally from the Gulf of Aqaba (***55***; 29° N) to New Caledonia (***27***; 22° S).	Some authors (e.g. Debenay 2012) regard this species as synonymous with *Planostegina longisepta* (Zheng).

Supplementary Table S3: List of synonymized species records. References and corresponding figures are given; nf = not figured.

Study	Initial species identification	Figures	Synonomized to
Albani 1968	*Sorites marginalis* (Lamarck)	nf	*Sorites orbiculus* (Forskal)
Baccaert 1987	*Amphisorus hemprichii* (Ehrenberg)	pl. 31, figs 1-5	*Amphisorus sauronensis* Lee, Burnham & Cevasco
	Calcarina spengleri (Gmelin)	pl. 97, figs 3-5; pl. 98, figs 1, 2	*Calcarina hispida* Brady
	Nummulites cumingii (Carpenter)	pl. 105, figs 4-6	*Nummulites venosus* (Fichtel & Moll)
	Peneroplis pertusus (Forskal)	pl. 15, figs 3, 4	*Dendritina striatopunctata* Hofker
	Sorites discoideus (Flint)	pl. 21: 2, 3; pl. 22: 1, 2; pl. 23: 1; pl. 24: 1	*Laevipeneroplis bradyi* (Cushman)
Bicchi *et al.* 2002	*Sorites discoideus* (Flint)	pl. 4, fig. 5	*Parasorites orbitolitoides* (Hofker)
Brady 1884	*Alveolina boscii* Defrance	pl. 17, figs 7-12	*Alveolinella quoyi* (d'Orbigny)
	Alveolina melo Fichtel & Moll	pl. 17, figs 13-15	*Borelis pulchra* (d'Orbigny)
	Calcarina spengleri Linné	pl. 108, fig. 5	*Calcarina gaudichaudii* d'Orbigny
	Calcarina spengleri Linné	pl. 108, fig. 7	*Calcarina mayori* Cushman
	Nummulites cumingii Carpenter	pl. 112, figs 11-13	*Nummulites venosus* (Fichtel & Moll)
	Orbitolites complanata var. *laciniata* Brady	pl. 16, figs 8-11	*Marginopora vertebralis* Quoy & Gaimard
	Peneroplis arietinus Batsch	pl. 13, figs 18, 19, 22	*Coscinospira hemprichii* (Ehrenberg)
	Peneroplis cylindraceus Lamarck	pl. 13, figs 20, 21	*Monalysidium acicularis* (Batsch)
Betjeman 1969	*Alveolinella* (*Alveolina*) *boscii* Defrance	nf	*Alveolinella quoyi* (d'Orbigny)
Cann & Clarke 1993	*Marginopora vertebralis* Quoy & Gaimard	fig. 3	*Amphisorus hemprichii* Ehrenberg
Chapman 1900	*Alveolinella* (*Alveolina*) *boscii* Defrance	nf	*Alveolinella quoyi* (d'Orbigny)
	Calcarina hispida Brady var. *pulchella* n. sp.	pl. 1, fig. 10	*Calcarina exuberans* Debenay
	Heterostegina depressa d'Orbigny (microspheric B form)	pl. 3, fig. 6	*Planoperculina heterosteginoides* (Hofker)
	Orbitolites complanata Lamarck	nf	*Marginopora vertebralis* Quoy & Gaimard
	Peneroplis (*Monalysidium*) *cylindraceus* (Lamarck)	nf	*Monalysidium acicularis* (Batsch)
Chapman & Parr 1938	*Operculina ammonoides* Gronovius forma *gaimardi*	p.290, pl.17, figs 14-16	*Assilina gaimardi* (d'Orbigny)
Cheng & Zheng 1978	*Peneroplis pertusus* (Forskal)	pl. 14, figs 8-14; pl. 15, figs 1, 2	*Peneroplis planatus* (Fichtel & Moll)
Cushman 1917	*Peneroplis pertusus* (Forskal) var. *arietinus* (Batsch)	pl. 36, fig. 2; pl. 37, fig. 5	*Coscinospira hemprichii* Ehrenberg
	Peneroplis pertusus (Forskal) var. *carinatus* d'Orbigny	pl. 37, fig. 4	*Dendritina* cf. *D. zhengae* Hatta
	Orbitolites duplex Carpenter	pl. 38, figs 3, 4; pl. 39, fig. 1	*Amphisorus hemprichii* Ehrenberg
	Orbitolites complanata Lamarck	pl. 39, fig. 2	*Marginopora vertebralis* Quoy & Gaimard
	Alveolina boscii (Defrance)	pl. 39, fig. 3	*Alveolinella quoyi* (d'Orbigny)
Cushman 1921	*Peneroplis carinatus* d'Orbigny	nf, p. 482 (s. 1917)	*Dendritina* cf. *D. zhengae* Hatta
	Peneroplis cylindraceus (Lamarck)	p. 483	*Monalysidium acicularis* (Batsch)
	Alveolinella (*Alveolina*) *boscii* Defrance	p. 487: 99	*Alveolinella quoyi* (d'Orbigny)
	Orbitolites complanata Lamarck	p. 487, nf	*Marginopora vertebralis* Quoy & Gaimard
	Orbitolites complanata Lamarck var. *plicata* (Dana)	p. 487, nf	*Marginopora vertebralis* Quoy & Gaimard
	Orbitolites duplex Carpenter	p. 485, nf	*Amphisorus hemprichii* Ehrenberg
	Siderolites tetraedra (Gümbel)	pl. 75, fig. 5; pl. 76, figs 1-5	*Baculogypsinoides spinosus* Yabe & Hanzawa
	Calcarina baculatus (?) (Montfort)	pl. 75, fig. 3	*Calcarina mayori* Cushman
Cushman 1924	*Orbitolites duplex* Carpenter	p. 71, nf	*Amphisorus hemprichii* Ehrenberg

Supplementary Table S3 continued.

Study	Initial species identification	Figures	Synonomized to
Cushman 1933	*Cycloclypeus guembelianus* Brady	pl. 18, fig. 1	*Cycloclypeus carpenteri* Brady
	Operculina gaimardi d'Orbigny	pl. 13, figs 1-5	*Assilina discoidalis* (d'Orbigny)
	Operculina granulosa Leymerie	pl. 14, figs 1-7; pl. 15, figs 1-6; pl. 16, figs 1-3	*Assilina ammonoides* (Gronovius)
Cushman *et al.* 1954	*Spirolina arietina* (Batsch)	pl. 87, figs 4, 5	*Peneroplis pertusus* (Forskal)
	Peneroplis sp.	pl. 87, fig. 3	*Dendritina zhengae* Ujiié in Hatta & Ujiié
	Calcarina hispida Brady	pl. 90, figs 9-12; pl. 92, figs 3-7	*Calcarina mayori* Cushman
	Marginopora vertebralis Blainville	pl. 82, figs 5, 6	*Amphisorus kudakajimaensis* (Gudmundsson)
Debenay 2012	*Borelis schlumbergeri* (Reichel)	p. 104, 281	*Borelis pulchra* (d'Orbigny)
Fajemila & Langer 2015	*Borelis schlumbergeri* (Reichel)	fig. 2: 3	*Borelis pulchra* (d'Orbigny)
	Monalysidium confusa McCulloch	fig. 2: 10	*Monalysidium acicularis* (Batsch)
Gabrié & Montaggioni 1982	*Marginopora*	nf	*Amphisorus hemprichii* Ehrenberg
Graham & Militante 1959	*Amphistegina radiata* (Fichtel & Moll) var. *venosa* (Fichtel & Moll)	p. 105, pl. 16, fig. 15	*Amphistegina lessonii* d'Orbigny
	Calcarina spengleri (Gmelin)	p. 107, pl. 17, figs 8-13	Fig. 8: *Calcarina mayori* Cushman; Fig. 10: *C. gaudichaudii* d'Orbigny; Fig. 12: *C. defrancei* d'Orbigny; Fig. 13: *C. hispida* Brady
	Calcarina hispida Brady	p. 106, pl. 17, figs. 6, 7	*Calcarina mayori* Cushman
	Operculina complanata (Defrance)	pl. 12, fig. 5	*Assilina bartschi* (Cushman)
	Marginopora vertebralis Quoy & Gaimard	pl.9, figs 19, 20	*Amphisorus hemprichii* Ehrenberg
	Spirolina arietina (Batsch)	pl.10, figs 9-11	*Coscinospira hemprichii* Ehrenberg
	Peneroplis sp.	pl. 10, figs 5, 6	*Dendritina zhengae* Ujiié in Hatta & Ujiié
	Dendritina antillarum Orbigny	pl. 9, fig. 17	*Dendritina ambigua* (Fichtel & Moll)
	Dendritina antillarum Orbigny	pl. 9, fig. 18	*Dendritina striata* Hofker
	Sorites discoideus (Flint)	pl. 9, fig. 22	*Laevipeneroplis malayensis* (Hofker)
	Neoalveolina pulchra (d'Orbigny)	pl. 10, fig. 13	*Alveolinella quoyi* (d'Orbigny)
Haig 1988	*Peneroplis pertusus* (Forskal)	pl. 9, fig. 22	*Peneroplis planatus* (Fichtel & Moll)
	Sorites marginalis (Lamarck)	pl. 9, figs 20, 21	*Sorites orbiculus* (Forskal)
Haig 1997	*Nummulites venosus* (Fichtel & Moll)	nf (Syn: L&T 1994)	*Nummulites cumingii* (Carpenter)
	Peneroplis pertusus (Forskal)	nf (Syn: L&T 1994)	*Peneroplis planatus* (Fichtel & Moll)
Hallock 1984	*Marginopora vertebralis* (Blainville)	fig. 1: 4	*Amphisorus hemprichii* Ehrenberg
	Peneroplis proteus d'Orbigny	fig. 1: 1	*Laevipeneroplis malayensis* (Hofker)
Hallock 1977	*Spirolina arietina* (Batsch)	pl. 3, fig. 4	*Coscinospira hemprichii* (Ehrenberg)
	Archaias angulatus (Fichtel & Moll)	pl. 3, fig. 1	*Laevipeneroplis malayensis* (Hofker)
	Marginopora vertebralis (Blainville)	pl. 3, fig. 2	*Amphisorus hemprichii* Ehrenberg
	Peneroplis pertusus (Forskal)	pl. 3, fig. 3	*Peneroplis planatus* (Fichtel & Moll)
Hatta & Ujiié 1992	*Borelis pulchra* (d'Orbigny)	p. 77, pl. 15, fig. 1	*Alveolinella quoyi* (d'Orbigny)
	Calcarina "spengleri" (Gmelin)	p. 202, pl. 48, figs 1-5	*Calcarina mayori* Cushman
	Operculina ammonoides (Gronovius)	pl. 50, fig. 7	*Assilina gaimardi* (d'Orbigny)
	Dendritina striata Hofker	pl. 15, fig. 6	*Dendritina ambigua* (Fichtel & Moll)
	Peneroplis pertusus (Forskal)	pl. 16, fig. 1	*Peneroplis arietinus* (Batsch)

Supplementary Table S3 continued.

Study	Initial species identification	Figures	Synonomized to
Hatta & Ujiié 1992	*Spirolina* sp.	pl. 16, fig. 5	*Monalysidium okinawaensis* (Ujiié & Hatta)
	Dendritina striata Hofker	pl. 15, fig. 4	*Dendritina striatopunctata* Hofker
	Peneroplis planatus (Fichtel & Moll)	pl. 15, fig. 2	*Peneroplis antillarum* (d'Orbigny)
Haunold *et al.* 1997	*Operculina ammonoides*	fig. 14	*Nummulites venosus* (Fichtel & Moll)
Hayward *et al.* 1999	*Sorites marginalis* (Lamarck)	p. 108, pl. 6, figs 12, 13	*Sorites orbiculus* (Forskal)
Heron-Allen & Earland 1914/15	*Peneroplis cylindraceus* (Lamarck)	nf	*Monalysidium acicularis* (Batsch)
	Orbitolites marginalis Lamarck	nf	*Sorites orbiculus* (according to text)
	Orbitolites duplex Carpenter	nf	*Amphisorus hemprichii* Ehrenberg
	Orbitolites complanata Lamarck	nf	*Amphisorus hemprichii* Ehrenberg
	Alveolina boscii (Defrance)	nf	*Borelis schlumbergeri* (Reichel)
	Alveolina melo (Fichtel & Moll)	nf	*Borelis pulchra* (d'Orbigny)
Hofker 1927	*Baculogypsina tetraedra* (Gümbel)	pl. 22, figs 1-5; pl. 23, figs 2-7,	*Baculogypsinoides spinosus* Yabe & Hanzawa
Hofker 1930	*Archaias discoideus* (Flint)	pl. 56	*Laevipeneroplis malayensis* (Hofker)
Hofker 1933	*Orbitolites duplex* Carpenter	nf	*Amphisorus hemprichii* Ehrenberg
	Archaias discoideus (Flint)	nf	*Laevipeneroplis malayensis* (Hofker)
Hofker 1950	*Dendritina carinata* d'Orbigny	p. 230, text figs 6-9	*Dendritina* cf. *D. zhengae* Hatta
Hohenegger 1994	*Calcarina hispida* Brady	fig. 6: 5	*Calcarina quoyi* d'Orbigny
	Nummulites venosus (Fichtel & Moll)	fig. 7: 5	*Nummulites cumingii* (Carpenter)
Hohenegger 1996	*Laevipeneroplis proteus*	nf	*Laevipeneroplis malayensis* (Hofker)
Hohenegger 1999	*Calcarina hispida* Brady form *defrancii* d'Orbigny	fig. 24	*Calcarina mayori* Cushman
	Calcarina hispida Brady form *spinosa*	fig. 25	*Calcarina hispida* Brady
Holzmann *et al.* 2001	*Marginopora* cf. *M. kudakajimaensis* Gudmundsson	pl. 2, figs 5, 6	*Amphisorus kudakajimaensis* (Gudmundsson)
	Parasorites sp. A	pl. 2, figs 1, 2	*Parasorites orbitolitoides* (Hofker)
	Laevipeneroplis sp.	pl. 3, figs 12, 13	*Laevipeneroplis malayensis* (Hofker)
Kuwano 1956	*Amphistegina radiata* (Fichtel & Moll)	pl. 29, figs 4-9	*Amphistegina lessonii* d'Orbigny
	Marginopora vertebralis (Blainville)	pl. 28, figs 16-17	*Amphisorus hemprichii* Ehrenberg
	Borelis sp.	pl. 29, fig. 1	*Borelis schlumbergeri* (Reichel)
Langer *et al.* 2013	*Sorites variabilis* Lacroix	fig. 7: 21	*Parasorites orbitolitoides* (Hofker)
Le Calvez 1965	*Operculina mayottana* LeCalvez	pl. 15, fig. 7	*Assilina ammonoides* (Gronovius)
	Operculina granulosa Leymerie	pl. 16, fig. 2	*Assilina bartschi* (Cushman)
	Cycloclypeus guembelianus Brady	pl. 15, fig. 3	*Cycloclypeus carpenteri* Brady
	Amphistegina lessonii d'Orbigny var. *conoidea*	pl. 15, fig. 1	*Amphistegina lessonii* d'Orbigny
	Operculinella sp.	pl. 15, fig. 2	*Assilina ammonoides* (Gronovius)
	Amphistegina antillarum (d'Orbigny)	pl.16, fig. 9	*Heterostegina depressa* d'Orbigny
	Operculinella cummingii Carpenter	pl. 15, fig. 4	*Nummulites venosus* (Fichtel & Moll)
Levy *et al* . 1996	*Spirolina arietina* (Batsch)	nf	*Coscinospira hemprichii* Ehrenberg
	Calcarina spengleri (Gmelin)	pl. 2, figs 10, 12	*Calcarina defrancei* d'Orbigny
Lessard 1980	*Alveolina melo* (Fichtel & Moll)	nf	*Borelis pulchra* (d'Orbigny)
	Archaias angulatus (Fichtel & Moll)	nf	*Laevipeneroplis malayensis* (Hofker)
Lobegeier 2002	*Calcarina spengleri* (Gmelin)	pl. 1, figs 1-8; pl. 2, figs 1-7	*Calcarina hispida* Brady

Supplementary Table S3 continued.

Study	Initial species identification	Figures	Synonomized to
Loeblich & Tappan 1994	*Dendritina striata* Hofker	pl. 108, figs 5-10	*Dendritina striatopunctata* Hofker
	Spirolina arietina (Batsch)	pl. 108, figs 11, 12	*Coscinospira hemprichii* Ehrenberg
	Calcarina hispida Brady	pl. 375, figs 3-6	*Calcarina mayori* Cushman
	Calcarina mayori Cushman	pl. 376, figs 4-7	*Calcarina spengleri* (Gmelin)
	Nummulites venosus (Fichtel & Moll)	pl. 388, figs 5-9	*Nummulites cumingii* (Carpenter)
	Peneroplis pertusus (Forskal)	pl. 110, figs 1-5	*Peneroplis planatus* (Fichtel & Moll)
	Sorites marginalis (Lamarck)	pl. 112, figs 4, 5	*Parasorites orbitolitoides* (Hofker)
Makled & Langer 2011	*Sorites variabilis* Lacroix	fig. 8: 23-25	*Sorites orbiculus* (Forskal)
	Calcarina hispida Brady	fig. 10: 3-7	*Calcarina mayori* Cushman
	Sorites orbiculus (Forskal)	fig. 8: 26, 27	*Parasorites orbitolitoides* (Hofker)
	Peneroplis planatus (Fichtel & Moll)	fig. 8: 10-12	*Peneroplis arietinus* (Batsch)
	Monalysidium sp. A (Batsch)	fig. 8: 6, 7	*Dendritina zhengae* Ujiié in Hatta & Ujiié
McCulloch 1977	*"Operculina"* Species A	pl. 100, fig. 2	*Assilina ammonoides* (Gronovius)
	Dendritina pacifica , new species	pl. 100, fig. 4	*Dendritina striatopunctata* Hofker
	"Operculina" cf. *bartschi* Cushman	pl. 99, fig. 1	*Assilina bartschi* (Cushman)
	"Operculina" cf. *bartschi ornata* Cushman	pl. 99, fig. 2	*Assilina gaimardi* (d'Orbigny)
	"Operculina" cf. *elegans* Cushman	pl. 99, fig. 3	*Assilina elegans* (Cushman)
	"Operculina" bartschi plana Cushman	pl. 99, fig. 4	*Assilina bartschi* (Cushman)
	Spirolina arietina (Batsch)	pl. 100, figs 10, 11, 16	*Coscinospira hemprichii* Ehrenberg
	Dendritina alworthi , new species	pl. 100, fig. 5	*Dendritina ambigua* (Fichtel & Moll)
	Amphistegina cf. *lessonii* d'Orbigny	pl. 153, fig. 1	*Amphistegina radiata* (Fichtel & Moll)
	Amphistegina cf. *madagascariensis* d'Orbigny	pl. 154, figs 8, 9	*Amphistegina madagascariensis* d'Orbigny
	Monalysidium (?) cf. *politum* Chapman	pl. 100, fig. 15	*Euthymonacha polita* (Chapman)
	Baculogypsina cf. *sphaerulata* (Parker & Jones)	pl. 155, fig. 4	*Baculogypsina sphaerulata* (Parker & Jones)
	Calcarina guamensis , new species	pl. 155, fig. 8	*Calcarina defrancei* d'Orbigny
	Sorites cf. *marginalis* (Lamarck)	pl. 101, figs 1, 4-6	*Parasorites orbitolitoides* (Hofker)
	Marginopora cf. *vertebralis* Quoyi & Gaimard	pl. 101, fig. 10	*Marginopora vertebralis* Quoy & Gaimard
	Borelis clarionensis , new species	pl. 101, fig. 11	*Borelis schlumbergeri* (Reichel)
	Borelis cf. *melo* (Fichtel & Moll)	pl. 101, figs 13, 14	*Borelis pulchra* (d'Orbigny)
	Baculogypsinoides cf. *spinosus* Yabe & Hanzawa	pl. 155, figs 1-3	*Baculogypsinoides spinosus* Yabe & Hanzawa
	Peneroplis cf. *pertusus* (Forskal)	pl. 100, figs 8, 17	*Peneroplis antillarum* d'Orbigny
	Calcarina cf. *rustica* Todd & Post	pl. 155, fig. 6	*Calcarina* sp. (s.a. Debenay p. 189: *Calcarina* sp. 1)
Möbius 1880	*Alveolinella (Alveolina) boscii* Defrance	pl. 3, figs 13-15; pl. 4, fig. 1	*Borelis schlumbergeri* (Reichel)
	Alveolina melo Fichtel & Moll	pl. 4, figs 2, 3	*Borelis pulchra* (d'Orbigny)
	Heterostegina curva Möbius	pl. 13	*Heterostegina depressa* d'Orbigny
	Orbitolites complanata Lamarck ("simple type" of Carpenter 1862)	pl. 4, figs 4, 5; pl. 5, figs 1-4	*Amphisorus hemprichii* Ehrenberg
	Rotalia defrancei d'Orbigny	pl. 14	*Neorotalia calcar* (d'Orbigny)
Montaggioni 1981	*Alveolinella (Alveolina) boscii* Defrance	nf	*Borelis schlumbergeri* (Reichel)
Murray 1965, 1966	*Spirolina arietina* (Batsch)	nf	*Coscinospira hemprichii* Ehrenberg
Narayan & Pandolfi 2010	*Peneroplis pertusus* (Forskal)	pl. 1, fig. 29	*Peneroplis antillarum* d'Orbigny

Supplementary Table S3 continued.

Study	Initial species identification	Figures	Synonomized to
Nobes *et al.* 2008	*Calcarina spengleri* (Gmelin)	fig. 22s-v	*Calcarina defrancei* d'Orbigny
Parker 2009	*Peneroplis pertusus* (Forskal)	fig. 108a-d	*Dendritina striatopunctata* Hofker
	Peneroplis pertusus (Forskal)	fig. 108e-h	*Dendritina striata* Hofker
	Peneroplis pertusus (Forskal)	fig. 109a-d	*Peneroplis planatus* (Fichtel & Moll)
Parker & Gischler 2011	*Calcarina* sp. 1	pl. 6, figs 5-15	*Calcarina defrancei* d'Orbigny
Renema 2002	*Laevipeneroplis proteus* (d'Orbigny)	nf	*Laevipeneroplis malayensis* (Hofker)
Renema 2003	*Schlumbergerella neotetraedra* (Tobler)	figs 25, 26	*Schlumbergerella floresiana* (Schlumberger)
Renema *et al.* 2001	*Calcarina gaudichaudii* d'Orbigny	fig. 15: d	*Calcarina spengleri* (Gmelin)
	Dendritina ambigua (Fichtel & Moll)	fig. 7: c	*Dendritina striata* Hofker
	Laevipeneroplis sp.	fig. 7e, f	*Laevipeneroplis malayensis* (Hofker)
	Spirolina arietina (Batsch)	nf	*Coscinospira hemprichii* Ehrenberg
Rhumbler 1906	*Orbitolites duplex* Carpenter	tf. 4: 41, 42, 44-50	*Amphisorus hemprichii* Ehrenberg
Smith 1995	*Calcarina spengleri* (Gmelin)	fig. 3	*Calcarina gaudichaudii* d'Orbigny
Todd 1961	*Calcarina spengleri* (Gmelin)	pl. 25, figs 8, 9	*Calcarina defrancei* d'Orbigny
Todd 1957	*Peneroplis ellipticus* d'Orbigny	pl. 89, fig. 1	*Dendritina ambigua* (Fichtel & Moll)
	Calcarina spengleri (Gmelin)	pl. 91, fig. 11	*Calcarina defrancei* d'Orbigny
	Spirolina arietina (Batsch)	pl. 89, fig. 4	*Monalysidium acicularis* (Batsch)
	Spirolina arietina (Batsch)	pl. 89, fig. 3	*Peneroplis pertusus* (Forskal)
	Peneroplis proteus d'Orbigny	pl. 93, fig. 4	*Laevipeneroplis malayensis* (Hofker)
Todd 1965	*Amphistegina lessonii* d'Orbigny	pl. 11, fig. 4	*Amphistegina radiata* (Fichtel & Moll)
	Pararotalia ozawai (Asano)	pl. 9, fig. 2	*Neorotalia calcar* (d'Orbigny)
	Amphistegina madagascariensis d'Orbigny (orange form)	pl. 11, fig 3	*Amphistegina lobifera* Larsen
	Calcarina hispida Brady	pl. 9, fig. 3	*Calcarina defrancei* d'Orbigny
van Marle	*Archaias angulatus* (Fichtel & Moll)	nf	*Laevipeneroplis malayensis* (Hofker)
Whittaker & Hodgkinson 1995	*Sorites marginalis* (Lamarck)	pl. 1, figs e, f	*Sorites orbiculus* (Forskal)
Yuvaraj *et al.* 2013	*Calcarina spengleri* (Gmelin)	pl. 2, figs 1-10	*Calcarina defrancei* d'Orbigny
Yuvaraja & Ramanujam 2013	*Coscinospira hemprichii* (Ehrenberg)	fig. 1: 5, 6	*Monalysidium acicularis* (Batsch)
	Peneroplis planatus (Fichtel & Moll)	fig. 1: 7	*Peneroplis arietinus* (Batsch)
Zapata & Olivares 2000	*Peneroplis proteus* Orbigny	fig. 66	*Parasorites orbitolitoides* (Hofker)
Zheng 1979	*Nummulites venosus* (Fichtel & Moll)	pl. 23, fig. 6	*Nummulites cumingii* (Carpenter)

Supplementary Table S4: Principle Component Analysis (PCA) factor loadings. Table listing the six PCs with Eigenvalues > 1 which were used for SDM development. Correlations > 0.6 are highlighted in bold face. Values between 0 and 1 indicate a positive contribution to the axis; values between -1 and 0 indicate a negative contribution.

Original variable	**PC1**	**PC2**	**PC3**	**PC4**	**PC5**	**PC6**
calcite	**-0.64262006**	0.16149725	-0.04342687	-0.3606214	0.01762901	-0.39973022
chlomax	**-0.86050505**	0.15288957	0.062403	0.1302453	0.22680541	0.36312702
chlomean	**-0.92283085**	0.20203705	0.01302902	-0.05514896	0.18725872	0.01121236
chlomin	**-0.80915779**	0.22663421	-0.03464818	-0.2189231	0.1308632	-0.31295006
chlorange	**-0.73761954**	0.10306694	0.08501338	0.2269541	0.22225012	0.53230058
cloudmax	0.11017937	**0.7442882**	-0.37264935	0.10591191	-0.05120987	0.10262059
cloudmean	0.25355607	**0.68725728**	**-0.61149549**	0.13290334	0.1392417	0.0126086
cloudmin	0.31751462	0.53023094	**-0.62679622**	0.1250213	0.28173945	-0.02631515
damax	**-0.92702134**	0.1747413	0.00455261	0.02597724	0.01210232	0.17562367
damean	**-0.94676231**	0.19577039	-0.02797937	-0.10893235	0.00969767	-0.04544569
damin	**-0.88871815**	0.21484455	-0.05376483	-0.22907874	0.02476888	-0.23839914
dissox	-0.20555459	**-0.71197689**	-0.49326157	0.12260854	0.05882059	-0.07091836
nitrate	-0.3366832	-0.22926277	0.17521134	**0.74657013**	-0.01846496	-0.18503158
parmax	-0.11567324	**-0.74302172**	0.46895558	-0.1342212	-0.05402519	0.02125362
parmean	0.06857304	0.16547738	**0.87503666**	0.08031803	0.03356193	-0.00515351
ph	0.29538832	-0.32971773	-0.13921172	-0.27434373	0.20548941	0.38493882
phos	-0.30527915	0.08070176	0.23152229	**0.74570033**	-0.09485578	-0.14566218
salinity	0.0524217	-0.57460123	0.40848542	-0.06071948	0.29579386	-0.01462818
silicate	-0.28592	0.21191986	-0.19144052	0.16430265	**-0.76308225**	0.06059119
sstmax	0.01461661	**0.69608989**	0.40415874	-0.25047214	-0.33955128	0.1878103
sstmean	0.22104269	**0.83042057**	0.46044399	-0.11782598	-0.05700742	0.04948867
sstmin	0.34956211	**0.78693852**	0.44760521	0.00283167	0.14367233	-0.05301959
sstrange	-0.45456565	-0.47945666	-0.26596433	-0.2091875	-0.47021665	0.22478421
**Eigenvalues**	6.74319413	5.22724258	3.10628674	1.73828613	1.34472777	1.07826431
Explained Variance	*29.3182354*	*22.7271416*	*13.5055945*	*7.55776577*	*5.84664248*	*4.6881057*

Supplementary Table S5: SDM performance for individual species. Information on the threshold (*Equal test sensitivity and specificity logistic threshold) applied, the number of training and test samples, the model performance and evaluation, and the variable contribution are given. Test AUC values indicate performance as follows: > 0.9 show very good, > 0.8 show good, and > 0.7 show useful discrimination ability of the model. High values in variable contribution are highlighted in bold face.

				Model performance		Variable contribution					
**Species**	**Threshold***	**Train. samples**	**Test samples**	**Train. AUC**	**Test AUC**	**PC1**	**PC2**	**PC3**	**PC4**	**PC5**	**PC6**
Alveolinella quoyi	0.4169	40	9	0.8738	0.7918	25.9251	12.8567	3.2135	**49.173**	6.6154	2.2163
Amphisorus hemprichii	0.4057	100	24	0.819	0.7867	19.2102	11.9139	15.3294	**42.7631**	10.2011	0.5823
Amphisorus kudakajimaensis	0.4748	6	1	0.8795	0.7092	**32.6061**	13.0766	10.5674	**30.992**	12.5763	0.1816
Amphisorus sauronensis	0.5647	4	1	0.9751	0.8578	3.4798	**33.1906**	6.0718	**26.8469**	**26.6827**	3.7282
Amphistegina bicirculata	0.438	24	6	0.9154	0.877	17.716	8.6338	8.2772	**41.5113**	14.5189	9.3429
Amphistegina lessonii	0.4069	132	32	0.8332	0.8081	17.6355	12.1314	14.6119	**42.5316**	11.0173	2.0723
Amphistegina lobifera	0.3968	62	15	0.8519	0.7943	**23.9388**	6.0785	**24.9433**	10.5869	**28.2264**	6.2262
Amphistegina madagascariensis	0.3964	35	8	0.9027	0.9079	10.7647	26.9943	**30.7949**	23.7531	4.3789	3.3141
Amphistegina papillosa	0.4328	31	7	0.8809	0.8666	16.1973	10.1029	7.266	**48.5256**	12.2707	5.6376
Amphistegina radiata	0.4035	86	21	0.8821	0.8626	19.198	16.7909	14.1462	**44.0537**	5.6703	0.1409
Assilina ammonoides	0.4364	82	20	0.8301	0.821	19.1763	10.1473	11.4147	**54.6632**	4.2962	0.3023
Assilina bartschi	0.256	37	9	0.9674	0.9281	8.573	17.3971	20.9857	**38.0285**	1.63	13.3856
Assilina complanata	0.3759	34	8	0.9139	0.8876	**25.1765**	**26.0222**	12.5846	**29.7665**	3.9138	2.5364
Assilina discoidalis	0.373	25	6	0.9447	0.9155	18.7767	10.9366	14.3553	**40.9427**	14.6169	0.3718
Assilina gaimardi	0.4342	16	3	0.984	0.9754	20.2834	11.5099	6.1011	**43.3985**	17.436	1.2711
Assilina philippinensis	0.3731	20	5	0.9687	0.9565	10.8448	16.8684	8.528	**47.7077**	5.3579	10.6932
Baculogypsina sphaerulata	0.4329	41	10	0.879	0.8561	11.3781	**34.9269**	14.2723	23.3392	14.4714	1.6121
Baculogypsinoides spinosus	0.3855	37	9	0.9304	0.9178	11.898	**34.8404**	8.8591	**38.4625**	5.7932	0.1468
Borelis pulchra	0.425	34	8	0.9078	0.8911	17.0988	10.9708	**36.8892**	15.933	6.8914	12.2168
Borelis schlumbergeri	0.3201	27	6	0.9027	0.8355	18.081	1.6243	13.6773	**45.4983**	20.5873	0.5318
Calcarina defrancei	0.3311	46	11	0.9255	0.8933	20.2119	24.2037	11.9735	**39.7921**	3.4397	0.379
Calcarina gaudichaudii	0.3806	18	4	0.9447	0.8932	16.8225	**36.2648**	15.5786	18.3255	12.2365	0.772
Calcarina hispida	0.4038	47	11	0.9345	0.9265	17.3942	21.0773	18.7491	**34.3972**	6.4904	1.8918
Calcarina mayori	0.2891	53	13	0.9295	0.9198	17.8716	16.005	19.2919	**42.1092**	2.5809	2.1415
Calcarina spengleri	0.3469	58	14	0.9139	0.8814	5.6686	**33.6835**	**29.6487**	**26.9329**	2.6017	1.4646
Coscinospira hemprichii	0.252	19	4	0.8777	0.7973	4.6469	22.3946	**33.2837**	20.4972	16.363	2.8147
Cycloclypeus carpenteri	0.4306	20	4	0.8458	0.6762	10.23	26.6692	11.2156	**35.6602**	12.5477	3.6774
Dendritina ambigua	0.5565	11	2	0.9447	0.936	1.9587	9.1387	1.9105	**67.8432**	16.9466	2.2023
Dendritina striata	0.4997	8	2	0.8715	0.7862	14.8292	**39.1282**	**27.7373**	12.7528	3.2475	2.3049
Dendritina striatopunctata	0.5272	7	1	0.9553	0.7259	25.9413	7.3897	3.9334	**59.2935**	2.5401	0.9018
Dendritina zhengae	0.6004	8	1	0.9256	0.8791	**28.7034**	21.0284	10.0244	**30.7552**	6.3816	3.107
Dendritina cf. *D. zhengae*	0.582	5	1	0.9133	0.7513	**25.3927**	**23.0125**	4.1166	10.939	**28.6011**	7.9381
Euthymonacha polita	0.3369	33	8	0.8717	0.84	24.8869	12.5183	10.2458	**36.1131**	15.1414	1.0944
Heterocyclina tuberculata	0.708	7	1	0.9505	0.9571	23.8217	3.4599	4.1135	**56.7893**	10.6372	1.1783
Heterostegina depressa	0.4524	142	35	0.8114	0.8008	23.1932	6.268	**28.438**	19.5398	18.1916	4.3693
Laevipeneroplis malayensis	0.4018	19	4	0.9216	0.8913	19.9352	**28.8131**	13.8334	**33.0673**	1.6094	2.7416
Marginopora vertebralis	0.4505	64	16	0.8283	0.7479	14.5496	8.8003	12.5164	**29.3129**	**32.494**	2.3268
Monalysidium acicularis	0.3273	45	11	0.8429	0.7496	15.1658	4.4818	12.5423	**61.4453**	4.5072	1.8576
Monalysidium okinawaensis	0.5734	8	1	0.9162	0.8852	7.4216	11.295	11.7362	**44.6681**	18.9954	5.8837
Neorotalia calcar	0.42	68	16	0.8566	0.8296	17.2136	10.102	8.8134	**57.1926**	5.5857	1.0928
Nummulites cumingii	0.4194	8	2	0.9414	0.8017	13.0878	2.4335	1.3902	**62.9074**	17.187	2.9941
Nummulites venosus	0.3397	36	9	0.9328	0.8909	22.2081	17.0257	7.8867	**40.7203**	10.9973	1.162
Parasorites orbitolitoides	0.4378	27	6	0.8876	0.7789	17.211	12.0376	**27.7056**	19.1956	12.2671	11.5831
Peneroplis antillarum	0.4079	19	4	0.9526	0.8581	14.4857	16.7394	14.6443	**31.233**	16.4296	6.4681
Peneroplis arietinus	0.4721	22	5	0.8377	0.8012	7.9959	16.2499	**34.4454**	**36.5833**	3.5452	1.1804
Peneroplis pertusus	0.3789	80	19	0.8619	0.8068	20.1804	17.0888	17.818	**28.0384**	11.0079	5.8665
Peneroplis planatus	0.352	79	19	0.8634	0.863	19.7596	7.1063	5.1708	**64.5414**	3.211	0.2109
Planoperculina heterosteginoides	0.4114	7	1	0.8497	0.5487	14.1796	14.7143	5.8193	18.6758	**41.192**	5.419
Planostegina operculinoides	0.46	12	3	0.9032	0.8308	21.1754	11.4425	4.175	20.5506	**40.841**	1.8156
Schlumbergerella floresiana	0.6799	8	2	0.975	0.9762	14.5968	**25.9904**	16.4777	14.2873	13.0217	15.626
Sorites marginalis	0.4545	57	14	0.8486	0.8023	10.5187	16.8184	**29.0156**	23.3285	16.7086	3.6103
Sorites orbiculus	0.3982	90	22	0.8731	0.864	**33.1194**	4.1176	20.4505	20.6369	18.3485	3.3271
